# HDAC inhibition potentiates anti-tumor activity of macrophages and enhances anti-PD-L1-mediated tumor suppression

**DOI:** 10.1038/s41388-020-01636-x

**Published:** 2021-02-09

**Authors:** Xiaolei Li, Xiao Su, Rui Liu, Yongsha Pan, Jiankai Fang, Lijuan Cao, Chao Feng, Qianwen Shang, Yongjing Chen, Changshun Shao, Yufang Shi

**Affiliations:** The First Affiliated Hospital of Soochow University, State Key Laboratory of Radiation Medicine and Protection, Institutes for Translational Medicine, Soochow University Medical College, Suzhou, 215123 Jiangsu China

**Keywords:** Cancer, Immunotherapy

## Abstract

Despite the widespread use of the blockade of immune checkpoints, for a significant number of cancer patients, these therapies have proven ineffective, presumably due to the immunosuppressive nature of the tumor microenvironment (TME). Critical drivers of immune escape in the TME include tumor-associated macrophages (TAMs) and myeloid-derived suppressor cells (MDSCs), which not only mediate immune suppression, but also facilitate metastatic dissemination and impart resistance to immunotherapies. Thus, strategies that convert them into tumor fighters may offer great therapeutic potential. In this study, we evaluated whether pharmacologic modulation of macrophage phenotype by HDAC inhibitors (HDACi) could produce an anti-tumor effect. We demonstrated that low-dose HDACi trichostatin-A (TSA) markedly reshaped the tumor immune microenvironment by modulating the suppressive activity of infiltrating macrophages and inhibiting the recruitment of MDSCs in various tumors. These actions, in turn, augmented anti-tumor immune responses and further enhanced anti-tumor effects of immunotherapies. HDAC inhibition, however, also upregulated PD-L1, thereby limiting the beneficial therapeutic effects. Indeed, combining low-dose TSA with anti-PD-L1 in this model significantly enhanced the durability of tumor reduction and prolonged survival of tumor-bearing mice, compared with the effect of either treatment alone. These data introduce HDAC inhibition as a potential means to harness the anti-tumor potential of macrophages in cancer therapy.

## Introduction

The success of checkpoint immunotherapy has created optimism that cancer may be curable. However, not all patients respond, resistance is common and many patients relapse owing to immune escape [1, 2]. Although multiple factors can contribute to the resistance of cancers to immunotherapies, one dominant player is the immunosuppressive myeloid cells present within the tumor tissues that can drive T cell exclusion and dysfunction [3]. As one of most abundantly infiltrating leukocyte populations in the tumor microenvironment (TME), macrophages significantly contribute to cancer progression by stimulating proliferation, angiogenesis, metastasis, and by creating an immune suppressive environment [4, 5]. Therefore, high numbers of tumor-associated macrophages (TAMs) often correlate with early local or metastatic relapse, leading to poor survival in patients with cancer [6, 7]. As TAMs profoundly blunt anti-tumor immunity, TAMs become increasingly targeted in clinical oncology [8–10]. Thus, even if using the blockade of immune checkpoints could mask the inhibitory receptor activation of T cells, the hostile local immunosuppressive TME created by TAMs-derived factors (i.e., arginase-1 (Arg1) and interleukin-10) directly reduces the cytotoxicity of T cells against cancer cells [11]. Strategies that deplete or stimulate TAMs have had some success [8, 9]. However, depleting TAMs may thus inherently suppress the overall phagocytic ability of macrophages in tumors [12]. This is concerning, as widely used antibody-based therapeutics require substantial phagocytic activity of macrophages to induce antibody-dependent cell cytotoxicity/phagocytosis (ADCC/ADCP) [13, 14]. Moreover, macrophages have been shown to be required for the efficacy of cancer immunotherapy [15], depleting TAMs during cancer therapy may undermine immunotherapy. Therefore, attempts have been made to repolarize macrophage phenotypes, instead of depleting them [8, 9]. A better understanding of mechanisms governing TAMs phenotypic changes and functional responses is fundamental to the development of cancer immunotherapies targeting macrophages.

Accumulating evidence indicates that epigenetic factors are involved in modulating the TME and regulating the anti-tumor immune response [16]. For instance, DNA methyltransferases and histone deacetylases (HDAC) can modulate anti-tumor immunity [17]. Several lines of evidence support that, beyond their potential as monotherapies, epigenetic drugs could act in synergy with other anti-cancer therapies [18, 19]. The rationale of employing epigenetic drugs is supported by finding that the increased presence of histone acetylation (e.g., H3K9 or H3K27 acetylation) and active chromatin state are tightly correlated with the function of CD8^+^ T cells [20]. The tumor-infiltrating lymphocytes (TILs) in immunosuppressive TME acquire a dysfunctional chromatin state in advanced tumor stages, which may limit the efficacy of immunotherapies [21, 22]. Thus, to achieve effective cancer immunotherapy, it is necessary to re-program the unresponsive T cells within the TME to regain their anti-tumor function. It is surprising that despite the importance of macrophages in cancer, epigenetic modifications in macrophages remain on the verge of being unraveled. Their changes in the context of ontogeny and the influence of environment cues under homeostasis reflect their ample plasticity [23]. Such macrophages plasticity is driven, in part, by epigenetic dynamics that can sustain stable phenotypes after activation. Innate immune memory (tolerance and training) in monocyte and macrophage lineages was shown to be mediated by specific changes to epigenetic modifications and thereby the metabolic and transcriptional programs of these cells [24]. As a consequence, it is conceivable that pro-inflammatory cytokine production, polarization and innate immune memory might be targeted at the epigenetic level. However, it remains unclear whether epigenetic modifications to innate cells, particularly macrophages, could modulate the immune responses to cancers in vivo.

In this study, we investigate the potential role of HDAC inhibition in alleviating immune suppression. We demonstrate that HDAC inhibition not only decreases the trafficking of myeloid-derived suppressor cells (MDSCs) into tumors, but also potentiates TAMs to specify anti-tumoral phenotype and bolster T cells activation within the TME. This in turn leads to reduced immune suppression and elevated overall anti-tumor immune response, which restrain tumor progression. However, in response to reduced immune suppression, programmed death-ligand 1(PD-L1) is upregulated on tumor cells and myeloid cells after HDACi treatment. Moreover, combination of low-dose TSA with anti-PD-L1 in syngeneic mouse tumor models results in an M1-like phenotype with diminished the immunosuppressive function of TAMs and promotes the overall anti-tumor immune response, leading to significantly reduced mouse tumor burden and a significant prolongation in survival compared with that in the control mice. These data suggest that reprogramming immunosuppressive myeloid cell responses via HDAC inhibition could potentially circumvent the limitations of current checkpoint blockade-based immunotherapy.

## Results

### Low-dose TSA inhibits tumor growth in syngeneic tumor models

Growing evidence demonstrates that HDACs are overexpressed in a variety of primary human tumors [25], including breast cancer, and we also found that higher levels of specific HDACs are associated with a significantly poorer outcome in patients with either breast cancer (Supplementary Fig. 1a) or lung cancer (Supplementary Fig. 1b), justifying the potential use of HDAC inhibitors (HDACi) in cancer therapy. To address the anti-cancer effect of trichostatin-A (TSA) as an HDAC inhibitor, we performed a series of experiments using multiple syngeneic mouse tumor models. Based on our previous study [26], we employed two concentrations, 0.5 μM/kg, referred to as low-dose, and 3 μM/kg, the dosage that was widely used for experimental tumor therapy, referred to as high-dose. We found that, regardless of dosage, both B16F10 and 4T1 tumor-bearing mice treated with TSA showed significantly reduced tumor burden and bore substantially smaller tumors than control mice given vehicle only (Supplementary Fig. 2a, b). Surprisingly, when similar studies were performed in nude mice, which are athymic, the therapeutic potency of low-dose TSA was abolished whereas that of high-dose TSA remained (Supplementary Fig. 2c), suggesting that the anti-cancer effects of low-dose TSA depends on an intact immune system. To further examine the direct cytotoxic effects of TSA on tumor cells, we treated various types of mouse cancer cell lines, including melanoma (B16F10), breast cancer (4T1) and lung cancer (LLC), with different concentrations of TSA ex vivo, and found that higher dosages of TSA do induce tumor cell death (Supplementary Fig. 3a, b). Since lower TSA dosages promoted tumor regression without targeting cancer cells directly, we adopted low-dose for subsequent experiments.

**Fig. 1:**
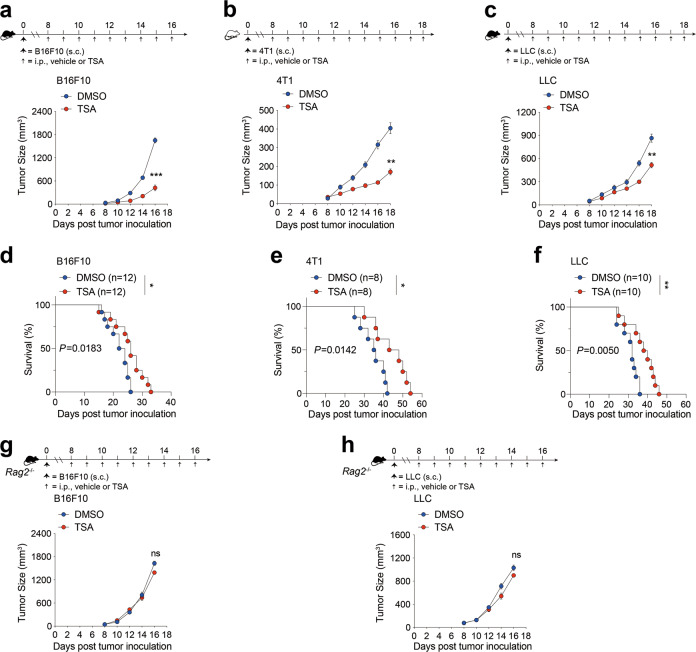
Low-dose TSA enables tumor growth inhibition that depends on T cells in syngeneic mouse models. **a** Timeline of mouse models of B16F10 melanoma with treatment schedules. S.C. subcutaneous, i.p. intraperitoneally. B16F10 tumor-bearing mice were injected with vehicle or low-dose TSA. Tumor size changes and the total tumor burden per mouse is shown. **b** Timeline of 4T1 tumor-bearing mice with treatment schedules. Average tumor growth and the total tumor burden per mouse were shown. **c** Timeline of LLC tumor-bearing mice with treatment schedules. LLC tumor-bearing mice were injected with vehicle or low-dose TSA and tumor size changes and the total tumor burden is shown. **d** Kaplan–Meier curves showing overall survival of B16F10 tumor-bearing mice treated with either vehicle or low-dose TSA. **e** Kaplan–Meier curves showing overall survival of mice that were challenged with 4T1 cells and given either vehicle or low-dose TSA. **f** Kaplan–Meier curves showing overall survival of LLC tumor-bearing mice treated with either vehicle or low-dose TSA. **g** and **h** B16F10 and LLC cells were implanted s.c. in RAG2 KO mice. Then, tumor-bearing mice were treated with either vehicle or low-dose TSA daily starting at day 8 after tumor inoculation. Tumor burden were assessed. Data are representative of at least three independent experiments. ns no significant. *P < 0.05, **P < 0.01. mean ± s.e.m.

To explore the potential role of TSA in potentiating the adaptive immune system, the therapeutic effect of low-dose TSA on tumor growth in the immunocompetent host was next evaluated. Toward this end, we subcutaneously implanted various mouse cancer cell lines, including melanoma (B16F10) (Fig. 1a), breast cancer (4T1) (Fig. 1b) and lung cancer (LLC) (Fig. 1c) into syngeneic mouse models. Starting on day 8 after tumor inoculation, we treated tumor-bearing mice with either vehicle or low-dose TSA until the mice were sacrificed. For each tumor type tested, administration of low-dose TSA delayed tumor progression (Fig. 1a–c), leading to a significantly decreased tumor burden in time point analysis and improved overall survival (Fig. 1d–f). The low-dose TSA was well tolerated as we observed no significant loss of body weight or other common toxic effect (data not shown). Lack of direct inhibitory effects of low-dose TSA on the growth or survival of tumor cells in vitro (Supplementary Fig. 3a, b) was consistent with our previous report [26]. Because TSA administered at such a low dose achieved an anti-tumor effect comparable to that with high-dose in xenograft models (3 μM/kg) in the syngeneic mouse models, we postulated that the anti-tumor effect of low-dose TSA in immunocompetent mice was not directly exerted on tumor cells. One possible explanation is that low-dose TSA might have an immunostimulatory role in vivo, which consequently affects tumor growth.

To determine the potential contribution of T lymphocytes in inhibiting tumor growth, we analyzed the efficacy of low-dose TSA in tumor-bearing recombination activating gene 2 (RAG2) knock out (KO) mice. RAG2-deficient mice lack the VDJ recombinase machinery necessary for rearranging antigen receptor genes, and as such do not produce mature B or T lymphocytes and are incapable of mounting an adaptive immune response. We subcutaneously transplanted either B16F10 tumor or LLC tumor into RAG2-deficient recipient mice. Indeed, low-dose TSA delivery failed to inhibit tumor growth in the RAG2-deficient recipient mice, indicating that adaptive immune cells are required for the anti-tumor responses elicited by low-dose TSA treatment (Fig. 1g, h). Taken together, these data suggest that addition of low-dose TSA elicited durable tumor remissions and markedly extended survival in tumor-bearing mice by conferring protective anti-cancer immunity.

### Low-dose TSA modestly increases anti-tumor T cell activity

**Fig. 2:**
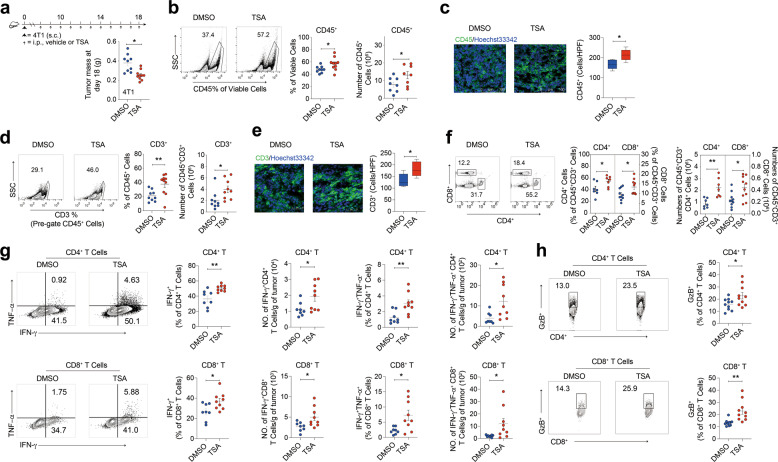
Low-dose TSA promotes TILs infiltration and their effector functions. **a** Mice were inoculated s.c. with 4T1 cells. Timeline of mouse models of tumors with treatment schedules. Tumor weights at the endpoint. **b** Representative dot plots and percentage of and the absolute number of CD45^+^ cells infiltration quantified by FACS. **c** Immunofluorescent staining of cryostat slides from tumors treated with either vehicle or low-dose TSA with anti-CD45 and Hoechst33342, (scale bar, 100 μm). **d** and **e** Representative dot plots and percentage of and the absolute number of CD3^+^ cells infiltration quantified by FACS and immunofluorescent staining of cryostat slides from tumors treated with either vehicle or low-dose TSA with anti-CD3 and Hoechst33342, (scale bar, 50 μm). **f** Percentage of CD4^+^ or CD8^+^ T cells in the CD3^+^ T cell population and the absolute number of CD4^+^ or CD8^+^ T cells in living cells. **g** Production of IFN-γ and TNF-α by CD4^+^ or CD8^+^ T cells was determined by intracellular cytokine staining. **h** Representative flow cytometry plots and bar graphs of granzyme B^+^ CD4^+^ T cells and granzyme B^+^ CD8^+^ T cells from TILs. Data are representative of at least three independent experiments. ns no significant. *P < 0.05, **P < 0.01. mean ± s.e.m.

The ability of low-dose TSA to promote tumor remissions as a single agent and its reliance on lymphocytes led us to speculate that HDAC inhibition alone might modulate endogenous anti-tumor T cell responses in TME, even in the absence of additional immunotherapy. To test this hypothesis, we analyzed the percentage and number of TILs from mice bearing either 4T1 or B16F10 tumors after vehicle or low-dose TSA treatment. TSA treatment resulted in a reduced 4T1 tumor mass when compared to vehicle (Fig. 2a). We observed an increase in the frequency and absolute number of CD45^+^ inflammatory cells in TILs from tumors after low-dose TSA treatment (Fig. 2b, c). The numbers of CD3^+^ T cells and their percentage in the total CD45^+^ population within tumors from mice given the low-dose TSA treatment were significantly higher than those from vehicle-treated mice (Fig. 2d, e). The number of tumor-infiltrating CD4^+^ T cells and CD8^+^ T cells and their percentage of the total T cells from mice given the low-dose TSA-treatment were also higher than those from mice given vehicle alone (Fig. 2f). Similarly, increased T cell infiltration was observed in subcutaneous B16F10 tumors in low-dose TSA-treated mice (Supplementary Fig. 4a), suggesting a general role for low-dose TSA in anti-tumor T cell responses. However, HDAC inhibition appeared only to promote T cell recruitment into tumors, as the systemic T cell content was not significantly altered in low-dose TSA-treated mice (Supplementary Fig. 4b). Meanwhile, administration of low-dose TSA in tumor-bearing mice did not result in significant changes in the frequency of Foxp3^+^ Treg cells in TILs, spleen and blood in tumor-bearing mice (Supplementary Fig. 4c). To determine whether T cell accumulation within tumors was the result of increased local T cell proliferation, we measured T cell proliferation by Ki-67 staining, but observed no significant differences in blood, spleen and TILs of mice treated with vehicle or low-dose TSA (Supplementary Fig. 4d). Similarly, low-dose HDACi treatment did not significantly affect proliferation of T cells isolated from spleens 3 days after anti-CD3/CD28 stimulation ex vivo (Supplementary Fig. 4e). These findings suggest that addition of low-dose TSA drives the trafficking of T cells to tumors, leading to a reinvigorated anti-tumor T cell response.

Next, we examined the effect of low-dose TSA on T cells functional activity within tumors. Two days after the last treatment, cells from tumors were briefly activated ex vivo with leukocyte activation mixture containing phorbol myristate acetate/ionomycin and then analyzed for cytokine production from T cells. Tumor-infiltrating CD4^+^ T cells and CD8^+^ T cells from mice given low-dose TSA treatment had an increased frequency of TNF-α and IFN-γ producers compared with vehicle treatment group (Fig. 2g). However, the frequencies of IFN-γ-producing CD4^+^ T cells and CD8^+^ T cells in the tumor-draining lymph nodes of mice were not altered by low-dose TSA treatment (Supplementary Fig. 4f). We also analyzed granzyme B expression, another marker of T cell activation. Compared with vehicle alone, addition of low-dose TSA increased the frequency of granzyme B-producing CD4^+^ T cells and CD8^+^ T cells in tumors (Fig. 2h). These data suggest that low-dose TSA only enhances the functional activity of tumor-infiltrating CD4^+^ T cells and CD8^+^ T cells but not that of draining lymph node resident T cells, suggesting that the immunostimulatory effect of low-dose TSA treatment was only limited to the TME.

### Addition of low-dose TSA specifies anti-tumoral phenotype of macrophages

Tumors escape immune surveillance by converting myeloid cells from anti-tumoral to tumor supportive and immunosuppressive state. We wanted to test the hypothesis that HDAC inhibition might inhibit the generation, recruitment or reprogramming of suppressive myeloid cells. In light of the robust generation of MDSCs in the 4T1 model, we examined the effect of low-dose TSA on MDSCs trafficking/accumulation. Consistent with the previous studies [27, 28], the percentages of MDSCs were markedly reduced amongst the tumor-infiltrating immune cells of low-dose TSA treated tumor-bearing mice compared to the control group in the tumors (Supplementary Fig. 5a). In addition, we also tested the ability of low-dose TSA to inhibit the recruitment of MDSCs into tumors in another immunotherapy resistant tumor model, the B16F10 melanoma model. Similarly, administration of low-dose TSA led to a decrease in the percentage of MDSCs in B16F10 tumor-bearing mice (Supplementary Fig. 5b). Overall these findings show that HDAC inhibition also leads to a decrease in MDSCs. However, importantly, both the percentage and the absolute number of TAMs per tumor weight did not change (Supplementary Fig. 5c), indicating that low-dose TSA did not reduce all types of myeloid cells within the TME, but rather diminishing MDSCs.

**Fig. 3:**
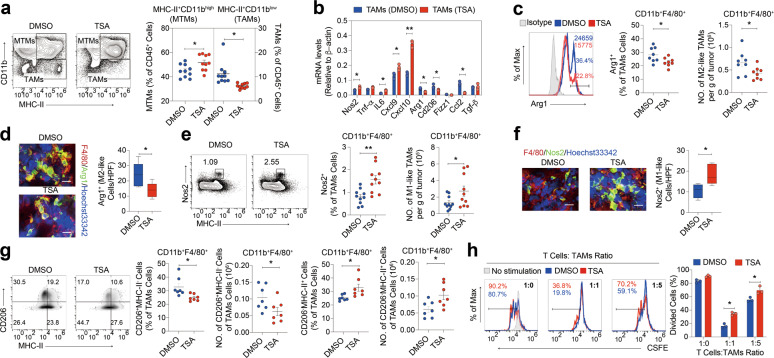
HDAC inhibition induces a pro-inflammatory conversion of TAMs. **a** Representative mammary tissue macrophages (MTMs) versus TAMs plots of MDSO-treated and low-dose TSA-treated mice with quantitation. **b** TAMs isolated from tumors of WT mice treated with DMSO control or low-dose TSA were assayed for the specific gene mRNA expression. **c** FACS of intracellular Arg1 staining in TAMs. **d** Representative immunofluorescent images of Arg1, F4/80 and Hoechst33342 in tumors of WT mice treated with DMSO control or low-dose TSA, (scale bar, 20 μm). **e** Representative dot plots and cumulative frequencies of M1-like macrophages. M1-like macrophage density as a cumulative absolute number of cells per gram of tumor. **f** Representative immunofluorescent images of Nos2, F4/80 and Hoechst33342 in tumors of WT mice treated with DMSO control or low-dose TSA, (scale bar, 20 μm). **g** FACS of intracellular staining of CD206 and MHC-II in infiltrating TAMs from tumors, the percentages of CD206^+^MHC-II^-^ or CD206^-^MHC-II^+^ TAMs and the absolute CD206^+^MHC-II^-^ or CD206^-^MHC-II^+^ TAMs numbers within tumors of WT mice treated with DMSO control or low-dose TSA. **h** T cells from macrophages and T co-culture system were subjected to suppress the activation of CSFE-labeled T cells. Data are representative of at least three independent experiments. ns no significant. *P < 0.05, **P < 0.01. mean ± s.e.m.

We next investigated the effect of low-dose TSA on the phenotype and function of the TAMs, which remained unchanged in abundance in response to low-dose TSA. Fluorescence-activated cell sorting (FACS) analysis of CD45^+^MHC-II^+^ cells from MMTV-PyMT tumors has been described to distinguish Notch-dependent, pro-tumor TAMs from homeostatic mammary tissue macrophages on the basis of the differential expression of CD11b (TAMs, CD11b^low^; mammary tissue macrophages, CD11b^high^) [29]. Accordingly, application of low-dose TSA resulted in a significant reduction in the proportion of pro-tumor TAMs gated this way (Fig. 3a and Supplementary Fig. 5d). We found that low-dose TSA led to significantly decreased mRNA expression levels of the M2 markers *Arg1*, *Cd206* and *Fizz1*, while the mRNA levels of nitric oxide synthase (*Nos2*) and *Il-6* associated with the M1-like phenotype were increased in TAMs (Fig. 3b). Intracellular staining and FACS analysis of tumors confirmed the reduction of Arg1 expression at protein level by low-dose TSA in TAMs (Fig. 3c). Arg1 downregulation in TAMs from low-dose TSA-treated group was further confirmed through immunofluorescence staining of dissociated tumors (Fig. 3d). Nos2 is one of the signature molecules expressed by M1 macrophages and is important for their anti-tumor function, therefore, we considered CD11b^+^Gr-1^-^F4/80^+^MHC-II^+^iNOS^+^ macrophages as M1 macrophages for our assessment. We observed an increase in frequency and total numbers of M1 macrophages within tumors from low-dose TSA-treated mice (Fig. 3e). Nos2 upregulation in macrophages from low-dose TSA-treated mice was further confirmed by immunofluorescence staining of dissociated tumors (Fig. 3f). The multi-ligand endocytic mannose receptor (CD206/MRC1) is an established marker of M2-like macrophages, whereas higher levels of MHC-II and/or co-stimulatory molecules serve as markers of more immunogenic M1-like macrophages. Thus, we analyzed the expression of these markers in TAMs isolated from vehicle- and low-dose TSA-treated tumors. As shown in Fig. 3g, intracellular staining and FACS analysis of tumors showed that addition of low-dose TSA led to a decrease in the frequency and total numbers of CD206^high^ MHC-II^low^ (M2-like macrophages) but an increase in the frequency and total numbers of CD206^low^ MHC-II^high^ (M1-like macrophages) in TAMs. Together, these results indicate that low-dose TSA may facilitate tumor regression by abrogating pro-tumoral TAM functions in mice, supporting the role of HDAC inhibition in enhancing a T cell-supportive TAM phenotype in vivo.

The ability of bone marrow-derived macrophage cells (BMDMs) treated with either vehicle or low-dose TSA to stimulate T cells was next assessed. Syngeneic T cells were isolated from the spleens of C57BL6/J mice, stained with CFSE, and subsequently co-cultured with BMDMs, in the presence of the anti-CD3/CD28. After 72 h stimulation ex vivo, T cell proliferation was assessed by FACS. We found that T cell proliferation was reduced when evaluated in the presence of M2-like BMDMs, correspondingly, concomitant addition of low-dose TSA in BMDMs reduced this effect (Supplementary Fig. 5e). Moreover, as expected, TAMs isolated from control treated tumors inhibited T cell proliferation in co-culture. However, TAMs isolated from tumors treated with low-dose TSA showed significantly reduced suppressive effects on T cell proliferation compared with that of the controls (Fig. 3h). Taken together, these data suggest that low-dose TSA modulates not only the accumulation/trafficking of MDSCs but also the suppressive capacity of TAM populations.

### Macrophages are essential for the anti-cancer efficacy of low-dose TSA therapy

**Fig. 4:**
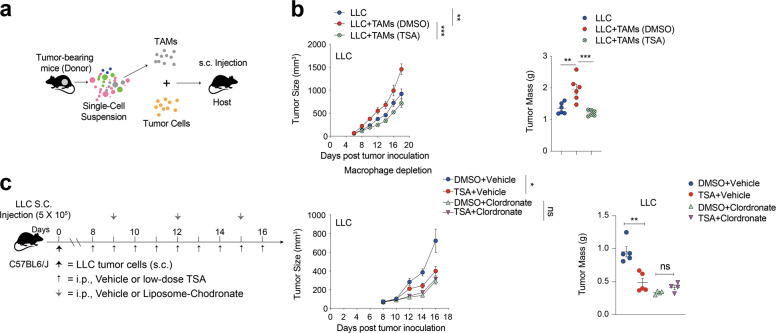
The anti-cancer efficacy of low-dose TSA is dependent upon macrophages. **a** Adoptive transfer method. **b** Tumor size changes and the total tumor burden per mouse resulting from tumor cells implanted together with TAMs (DMSO), TAMs (TSA) or no TAMs. Weights of tumors resulting from tumor cells implanted together with TAMs (DMSO), TAMs (TSA) or no TAMs. **c** Timeline of mouse models of LLC with treatment schedules. Tumor size changes and the total tumor burden per mouse, and weights of tumors resulting from tumors treated with DMSO + Vehicle, TSA + Vehicle, DMSO + clordronate and TSA + clordronate. Data are representative of at least three independent experiments. ns no significant. *P < 0.05, **P < 0.01. mean ± s.e.m.

Because HDAC inhibition stimulated pro-inflammatory responses in macrophages and enhanced anti-tumor immune responses to facilitate tumor regression, we speculated that the TAMs might act as mediators of the HDACi-elicited immunomodulatory activity. To definitively determine whether TAMs are responsible for HDACi-induced tumor protection and adaptive immune activation in vivo, we harvested TAMs from vehicle or low-dose TSA-treated LLC tumors and adoptively transferred the TAMs to new recipient WT mice together with LLC tumor cells (Fig. 4a). The addition of control TAMs accelerated tumor growth as compared with tumors injected with LLC alone. TAMs isolated from tumors treated with low-dose TSA co-injection significantly delayed tumor growth when compared with TAMs harvested from vehicle-treated group (Fig. 4b). These results indicate that delivery of low-dose TSA directly impairs the tumor-promoting activity of immunosuppressive macrophages, suggesting that the epigenetic rewiring of macrophages by HDAC inhibition may empower TAMs anti-tumor activities.

To further validate whether M1-like reprogramming of TAMs is responsible for the anti-tumor efficacy of low-dose TSA in mice, mice bearing pre-established tumors were treated with HDACi in combination with clodronate liposomes (Fig. 4c), which deplete macrophages from tissues [30]. As shown in Supplementary Fig. 5f, chlodronate administration resulted in about 5.5-fold reduction in tumor-infiltrating myeloid cells. Separate HDAC inhibitor and clodronate liposome treatment each partially inhibited tumor growth, but the combination had no additive effects (Fig. 4c). Taken together, these results provide evidence that the activated macrophages are required for the anti-tumor effect of HDAC inhibition.

### Low-dose TSA promotes M1-like macrophage polarization ex vivo

**Fig. 5:**
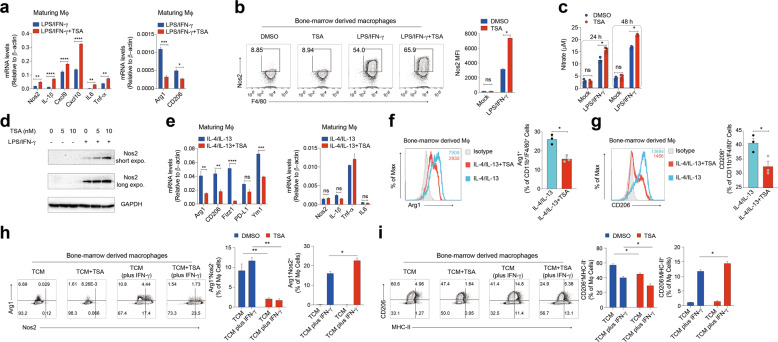
Low-dose TSA skews macrophages to M1-like phenotype ex vivo. **a** Maturing macrophages treated with IFN-γ (20 ng/ml) + LPS (100 ng/ml) in the presence or absence of TSA (10 nM) were assayed for the specific gene mRNA expression. **b**–**d** Maturing macrophages were stimulated with IFN-γ (20 ng/ml) + LPS (100 ng/ml) in the presence or absence of TSA (10 nM) to detect the expression levels of Nos2. **e** Maturing macrophages from IL-4 (20 ng/ml) + IL-13 (20 ng/ml) in the presence or absence of TSA (10 nM) were assayed for the specific gene mRNA expression. Maturing macrophages were treated with the indicated cytokines, the expression levels of Arg1 (**f**) and CD206 (**g**) were determined by FACS. **h** and **i** FACS of intracellular staining of CD206 and MHC-II or Arg1 and Nos2 macrophages pretreated with the regular medium (RM) or tumor-conditioned medium (TCM) in the presence or absence of TSA (10 nM) was determined. Data are representative of at least three independent experiments. *P < 0.05, **P < 0.01, ***P < 0.001, ****P < 0.0001, ns no significant.

The in vivo data demonstrated that HDAC inhibition altered TAM phenotypes and enabled T cells to acquire increased effector function. We next directly tested the impact of HDAC inhibition on polarization and effector activity of macrophages ex vivo. We generated BMDMs polarized to either a classic inflammatory or alternatively activated regulatory state. We found that administration of low-dose TSA had no direct effects on the growth or survival of macrophages in vitro (Supplementary Fig. 6a). We next stimulated primary mouse macrophages stimulated ex vivo with pro-inflammatory (IFN-γ, lipopolysaccharide (LPS)) or anti-inflammatory (IL-4, IL-13) conditions. Pro-inflammatory stimuli mostly upregulated the expression of innate immune cytokines (*Nos2*, *Tnf-α*, *Il1-β* and *Il6*), whereas anti-inflammatory stimuli mainly induced expression of immunosuppressive factors (*Arg1* and *Cd206*) similar to those expressed in TAMs. We further tested RNA expression of M1 and M2 markers in BMDMs after HDAC inhibition. Interestingly, when HDACi was added during the maturation of BMDMs, the resulting mature macrophages generally showed further increased expression of LPS/IFN-γ-regulated genes, such as *Nos2* and *Il1-β*, compared to levels in macrophages of controls (Fig. 5a, b, c, d). Apart from promoting M1-like macrophages polarization, low-dose TSA was shown to inhibit M2-like macrophages, as reflected by the decreased expression of prototypic M2 markers *Arg1* and *Cd206* (Fig. 5e, f, g). We also treated mature macrophages with low-dose TSA in pro-inflammatory or anti-inflammatory conditions. Similar to what was observed in maturing macrophages, the corresponding phenotypes were also observed in mature BMDMs exposed to low-dose TSA (Supplementary Fig. 6b, c, d, e). To corroborate these findings, we subsequently employed another HDACi, vorinistat (SAHA), and observed similar phenotypic changes (Supplementary Fig. 6f, g, h). Such phenotype switching was also confirmed by immunofluorescence analysis of macrophages under these conditions (Supplementary Fig. 6i–k). Taken together, these data revealed that HDAC inhibition could induce phenotypic changes in both maturing macrophages and existing mature macrophages.

Soluble factors derived from tumor cells can influence myeloid cell differentiation and promote the expansion and activity of TAMs and other immunosuppressive myeloid cells. To examine the direct effects of low-dose TSA on the responses of macrophages to tumor-derived factors, we treated BMDMs with regular media (RM) or tumor-conditioned media (TCM) from LLC tumor cells in the presence or absence of low-dose TSA, and then examined the protein expression levels of selected M1- or M2-like macrophages markers by FACS. As shown in Supplementary Fig. 6l, an increase in the percentage of M2-like macrophages was detected in BMDMs generated in the presence of TCM. These changes were significantly attenuated by TSA (Fig. 5h). In the presence of TCM, TSA-treated BMDMs expressed higher levels of MHC-II and lower levels of CD206 than controls (Fig. 5i). Collectively, these data suggest that addition of low-dose TSA results in the repolarization of macrophages towards pro-inflammatory polarization that could support anti-tumor T cell responses.

### HDAC inhibition and anti-PD-L1 have synergistic antitumorigenic effects

Our studies demonstrate that HDAC inhibition boosts immune responses by enhancing a pro-inflammatory phenotype of TAMs and reducing MDSCs. Moreover, we observed an increase in the tumor-infiltrating CD4^+^ T cells and CD8^+^ T cells from low-dose TSA treated tumor-bearing mice compared to control group. To investigate whether HDAC inhibition interacts with other immune therapies, we determined if HDAC inhibition could enhance the efficacy of checkpoint blockade in mouse tumor models. First, to determine the effect of HDAC inhibition on PD-L1 expression, we treated a panel of mouse tumor cell lines, including B16F10, 4T1 and LLC with HDACi for 48 h and analyzed the expression level of PD-L1. As PD-L1 must be expressed on the cell surface for successful targeting, we then assayed cell surface PD-L1 expression by FACS. Cell surface PD-L1 levels significantly increased following treatment with TSA in a dose dependent manner in all cell lines (Supplementary Fig. 7a, b, c). We also observed increased mRNA and protein expression of PD-L1 in both CD45^-^ and CD45^+^ cells in tumors from low-dose TSA-treated mice (Supplementary Fig. 7d, e). Because PD-L1 expression on host cells also affects the anti-tumor activity of anti-PD-L1 immunotherapy [31, 32], we investigated the effect of low-dose TSA on PD-L1 expression on immune cells, especially myeloid cells in the TME. An increase in the mRNA and protein expression of PD-L1 was observed on CD11b^+^ myeloid cells (Supplementary Fig. 7d, e). We thus reasoned that HDAC inhibition-based cancer treatment should be combined with PD-(L)1 blockade.

**Fig. 6:**
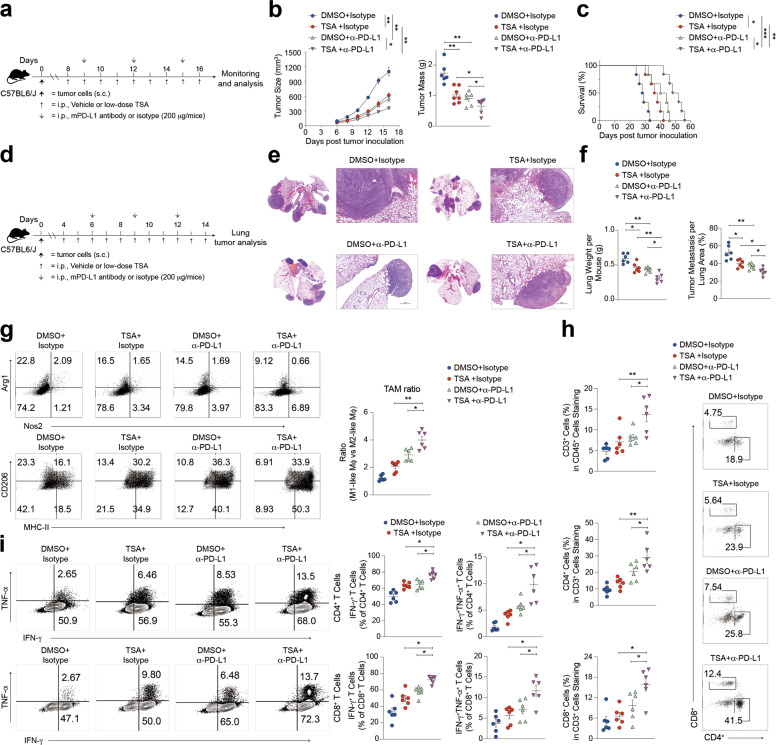
HDAC inhibition and anti-PD-L1 synergistically induce tumor regression. Mice were s.c. inoculated with tumor cells, tumors were removed at the endpoint. **a** Timeline of mouse models of tumor cells with treatment schedules. **b** Tumor size and weights of tumors resulting from tumor treated with either vehicle, TSA alone, anti-PD-L1, or anti-PD-L1 + TSA. **c** Kaplan–Meier curves showing overall survival of tumor-bearing mice treated with either vehicle, TSA alone, anti-PD-L1, or anti-PD-L1 + TSA. **d** Diagram depicting treated schedule for metastasis tumor model. **e** and **f** H&E staining of the lung sections at the end of the anti-tumor study. Scale bars, 500 mm. Quantification of tumor areas per lung section. **g** FACS of intracellular staining of CD206 and MHC-II or Arg1 and Nos2 in infiltrating TAMs from tumors treated with either vehicle, TSA alone, anti-PD-L1, or anti-PD-L1 + TSA. **h** Percentages of CD3^+^ T cells, CD4^+^ and CD8^+^ T cells within tumors treated with either vehicle, TSA alone, anti-PD-L1, or anti-PD-L1 + TSA were determined by FACS. **i** Single-cell suspensions from tumors treated at the indicated were stimulated with a cell stimulation cocktail and intracellular IFN-γ and TNF-α staining was performed. Representative dot plots and summarized data are shown. Cells were gated on CD4^+^ and CD8^+^ T cells. Data are representative of at least three independent experiments. *P < 0.05, **P < 0.01, ***P < 0.001, ns no significant.

To investigate whether HDAC inhibition acts in synergy with checkpoint-targeted therapies, we combined low-dose TSA and the checkpoint inhibitor anti-PD-L1 in mouse tumor models. To this end, mice were injected with tumor cells and treated on day 8 post injection with either vehicle, TSA alone, anti-PD-L1, or anti-PD-L1 + TSA (Fig. 6a). We found that while low-dose TSA and PD-L1 blockade each inhibited tumor growth and increased survival (Fig. 6b, c and Supplementary Fig. 7f), low-dose TSA and PD-L1 antibody in combination further reduced tumor growth and prolonged the overall survival compared to monotherapy groups in syngeneic mouse models (Fig. 6b, c and Supplementary Fig. 7f).

Next, we tested the anti-tumor activity of this combination therapy in an experimental metastasis lung cancer model. The tumor-bearing mice established with tail vein injection of tumor cells were also treated with either vehicle, TSA alone, anti-PD-L1, or anti-PD-L1 + TSA (Fig. 6d). We found that the metastases to the lungs were reduced in the combination treatment group (Fig. 6e, f and Supplementary Fig. 7g). Taken together, these data indicate that low-dose TSA in combination with anti-PD-L1 exerts a superior anti-tumor effect in this lung cancer model.

Anti-PD-L1 treatment has also been shown to promote myeloid cell activation in tumors, including M1-like TAM skewing [33]. We then asked whether low-dose TSA plus anti-PD-L1 could cooperatively foster immunostimulatory myeloid cell activation in tumors. FACS analysis of tumor-infiltrating immune cells showed that this combination therapy decreases the percentage of M2-like TAMs (CD206^high^MHC-II^low^ or Arg1^high^Nos2^low^) and increases the proportion of M1-like macrophages (CD206^low^MHC-II^high^ or Arg1^low^Nos2^high^) present in tumors and consequently increased the M1/M2 TAM ratio, compared to the other treatments (Fig. 6g and Supplementary Fig. 7h). To further determine whether sequential administration of low-dose TSA and PD-L1 blockade can improve T cell-mediated anti-tumor immunity by modulating the suppressive activity of infiltrating myeloid cells, we subsequently explored changes in T cell infiltration of tumors. Analysis of TILs shows that separate HDACi and PD-L1 inhibitor treatment each partially increased the recruitment of CD4^+^ and CD8^+^ T cells, and the combination of therapies further elevated these parameters (Fig. 6h). Since low-dose TSA alone could increase the effector function of both CD4^+^ and CD8^+^ T cells as measured by IFN-γ and TNF-α production (Fig. 2g), we wondered if the supplementation of anti-PD-L1 could further boost cytokine production by T cells. Indeed, in addition to increasing T cell frequency, low-dose TSA and PD-L1 blockade cooperatively enhanced IFN-γ and TNF-α production by both CD4^+^ and CD8^+^ T cells (Fig. 6i), suggesting that this combination therapy not only improved tumor infiltration by T cells but also enhanced their effector activation and promoted T-cell-mediated cytotoxicity. Taken together, these results showed that HDAC inhibition can synergize with T cell-targeted therapy to promote anti-tumor immune responses that induce tumor regression in syngeneic mouse models of cancer. This strongly suggests that combining HDAC inhibition with checkpoint blockade could yield additional anti-tumor activity, thereby providing a strong rationale to consider exploring HDAC inhibition to overcome resistance to immunotherapy in clinical trials.

## Discussion

**Fig. 7:**
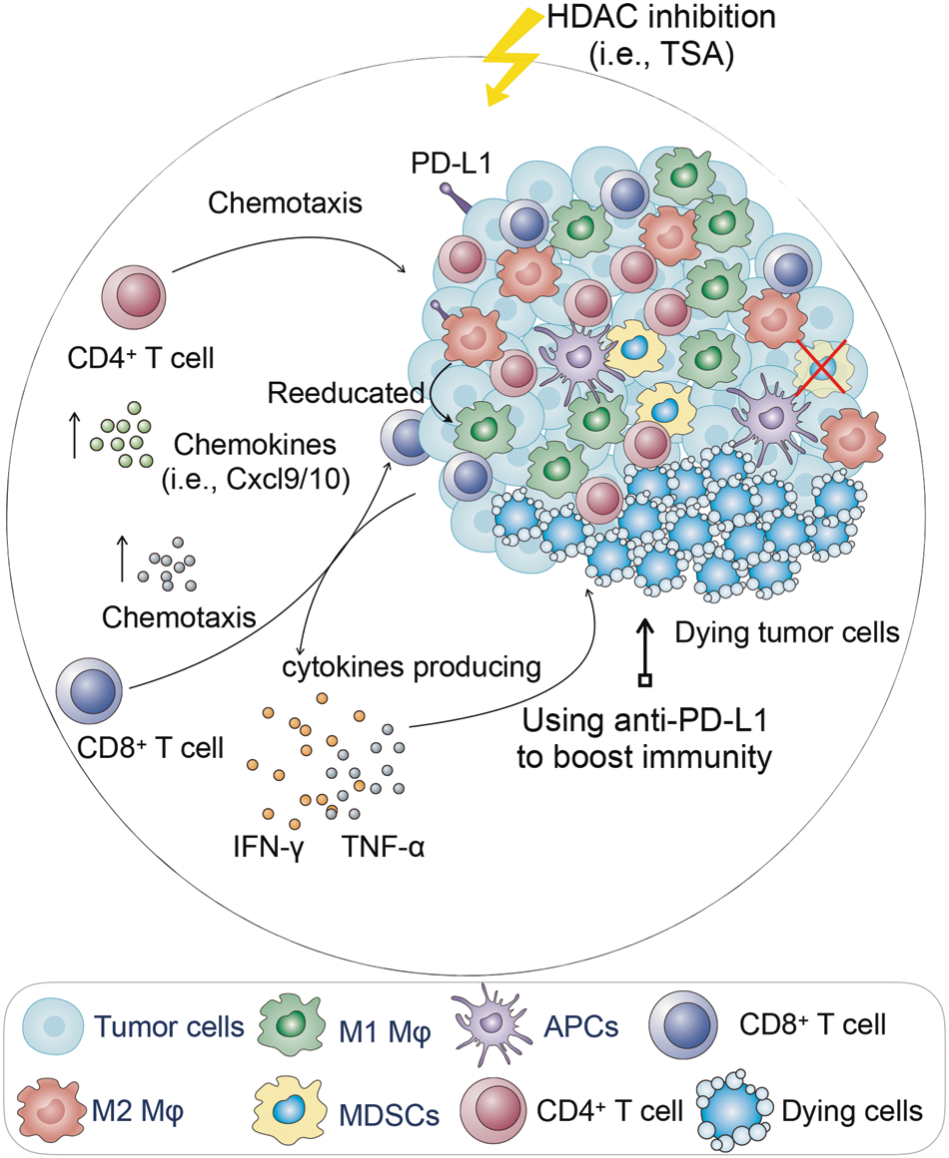
A model depicting the effect of HDAC inhibition on anti-tumor immunity. HDAC inhibition converts TAMs into pro-inflammatory macrophages that promote T cell responses to suppresses tumor growth. Low-dose TSA can inhibit the trafficking of MDSC into tumors. HDAC inhibition can also synergize with checkpoint-targeted therapy (i.e., PD-L1 antibody) to promote anti-tumor immune responses that induce tumor regression in syngeneic mouse models of cancer.

Immunotherapy shifted the paradigm of cancer treatment. Antibodies targeting the inhibitory T cell receptors CTLA-4 and PD-1 have significantly improved patient survival in many cases, but numerous cancers remain refractory to this approach. The cellular and molecular determinants of responsiveness versus resistance to immunotherapy are incompletely understood. Most of these immunomodulatory approaches have focused on manipulating the adaptive immune system. However, given that innate immunity plays a critical role in the induction and maintenance of adaptive immunity, a more effective therapeutic strategy should incorporate both arms of the immune system. Here, we report a previously undefined role of HDACi in inducing anti-cancer immunity in syngeneic mouse models. This effect is mediated through steering TAMs toward an anti-tumor phenotype and blocking the recruitment of MDSCs within tumors, which ultimately elicits a robust T cell-mediated anti-tumoral immune responses (Fig. 7). This finding has implications in cancer therapy since HDAC is a universal tumor epigenetic regulator and HDACi can potentially be applied to many other types of cancers. Moreover, sequential administration of low-dose TSA and anti-PD-L1 have synergistic anti-cancer effects in syngeneic mouse models, suggesting that the combination therapy may benefit PD-L1-resistant patients in the clinic.

Current thinking is that HDACi could kill tumor cells mainly by inhibiting tumor cell proliferation, inducing cell cycle arrest and facilitating tumor cell apoptosis [25]. Our results reveal a previously undefined role of TSA, an HDACi drug, in potentiating anti-tumor macrophages, and indicate that this drug also controls tumors by promoting anti-tumor immunity. By contrast, most previous studies use xenograft cancer models lacking an intact immune system. Only the tumor-intrinsic effects or part of the innate immune responses can be studied in xenograft models, which exclude T cell-mediated long-term protective immunity against tumor. In addition, most previous studies used high-dose or intensive dosing strategies that directly kill tumor cells more efficiently but actually dampen immune responses, either due to the toxicity to immune cells or the non-immunogenic death of tumor cells. Also, these intensive dosing strategies often lead to the emergence of tumor resistance mechanisms. Till date, only few reports have investigated the effects of HDACi on immune cells or its potential to be combined with checkpoint blockade-based immunotherapy. In this study, we took advantage of syngeneic mouse models with intact immune systems to fully evaluate the impact of lower doses with HDACi on host immune responses in tumor-bearing mice. This discovery that HDACi is an immune stimulatory drug might allow the design of better combinational treatments, including immunotherapy to amplify innate immunity.

Typically, macrophages are activated to undergo polarization into opposite phenotypes, classically activated or M1, associated with inflammation and immunity (anti-tumoral), and alternatively activated or M2, associated with repair and immune suppression (pro-tumoral) [8]. This nomenclature is an oversimplification because of macrophage plasticity in response to signals in the microenvironment and the diversity of phenotype falling between the two extremes, but reflects the counteracting activities [34]. TAMs, predominantly M2-like, have been considered “bad players”, correlating with poor prognosis in cancer patients [9, 10]. By contrast, it is known that the local cytokine environment can dictate the functional reprogramming of TAMs by influencing M1/M2 activation. Thus, local inflammatory signals induced by HDACi may potentially polarize myeloid cell function in favor of a pro-inflammatory phenotype, which can offer an alternative approach to subverting TAM suppressiveness. This may explain why TAMs from HDACi treated mice showed enrichment for M1-related genes and was able to confer tumoricidal capability. Indeed, microenvironment signals, such as IFN-γ, can skew TAM polarization from M2 to M1 with tumor-suppressive and anti-angiogenic properties [35]. Consistently, MS-275, a class I HDACi, can favor anti-tumor myeloid polarization as a result of increased local production of IFN-γ to promote sustained tumor progression in the context of anti-tumor immunity [36]. Thus, low-dose TSA may have induced M1-like conversion of TAMs possibly because of HDACi-induced local production of IFN-γ within tumors. Further studies should be carried out to verify this possibility.

Malignant cells have a remarkable capacity to modulate the development of myeloid progenitors by their ability to secrete factors that act distally on the bone marrow and splenic myeloid progenitors, or proximally on monocytes recruited to the TME [37]. As the executor of both innate and adaptive immunity, TAMs have been implicated in immune suppression at TME [9, 10]. In addition to being immunosuppressive, pro-tumor TAMs contribute to abnormally leaky and branched tumor vasculature [8–10]. In contrast, anti-tumor macrophages are associated with anti-angiogenic mechanisms including vessel pruning and normalization, which can substantially enhance the therapeutic potency of other cancer therapy [9]. Typically, CD206^high^ TAMs show an M2-like phenotype, while CD206^low^ cells are M1-like polarized, and have higher anti-tumor activity [10]. Of note, our data indicate that in the presence of tumor-derived factors, more CD206^high^ and less CD206^low^ BMDM are generated ex vivo from bone marrow progenitor cells but HDAC inhibition in myeloid cells attenuates this process, leading to the generation of more M1-like and less M2-like macrophages compared with that of control. CD206 is expressed in many subsets of myeloid cells other than macrophages, including immature dendritic cells and monocytes [38]. Whether CD206 expression is correlated to differential activation status in these cell types remains unknown. Intriguingly, Tie2^+^ monocytes almost uniformly express CD206 [39]. It remains to be seen whether the loss of CD206^high^ tumor-infiltrating monocytes upon concomitant delivery of low-dose TSA involves the Tie2^+^ monocytes and/or affects tumor vasculature. Remarkably, one previous study has reported that TMP195, a class IIa HDACi, affects CD11b^+^ leukocytes and establishes an anti-tumor microenvironment with normalized vasculature [40], further supporting this hypothesis.

MDSCs also contribute to immune tolerance in the TME and consequently affect the efficacy of immunotherapies [41]. Consistent with previous studies [27, 28], our data demonstrate that HDACi leads to a marked decrease of MDSCs within the tumors. This could be due in part to downregulation of the expression of CCR2 on monocytic (M)-MDSCs by HDACi, leading to the reduced infiltration of M-MDSCs into tumors [28]. Thus, HDACi showed an effect similar to that of CCR2 inhibitors that could also impede invasion of MDSCs into tumors and enhance checkpoint blockade effect [42]. However, HDACi has targets other than CCR2, hence HDACi might be better than CCR2 inhibitors for cancer therapy. Although the direct effect of HDACi on CCR2 expression cannot be excluded, low-dose epigenetic drugs may affect in part the expression of CCR2 in M-MDSCs from bone marrow via the modulation of NF-κB pathway [43, 44]. Although CCR2 expression on the TAMs is also likely to be reduced, interestingly, low-dose HDACi treatment did not simply reduce the percentage and absolute numbers of TAMs within tumors. Rather, it promoted the generation of pro-inflammatory TAMs. Analogous with our findings, a recent study demonstrated that HDAC inhibition enhances M1 and reduces M2 phenotype [45]. This may be partly due to other tumor-derived factors also attract circulating monocytes to the tumor site and differentiate into TAMs, such as CSF1, CCL5, CXCL12 and CX3CL1. Potentially, addition of low-dose HDACi might upregulate these factors, resulting in no significant change in the levels of TAMs at the tumor site, and further research is warranted. Some studies have revealed contradictory roles HDACs in macrophage activation and inflammatory responses, for instance, TSA can diminish the production of key pro‐inflammatory cytokines by LPS‐stimulated macrophages, and further evidence indicate that these anti‐inflammatory effects of TSA are owing to inhibition of class I HDACs [46], and enhanced autophagy [47] in macrophages. These findings are inconsistent with our results and may be due to the different dosage as well as the duration of TSA stimulation. Thus, how TSA selectively modulates the pro-inflammatory and anti-inflammatory targets in macrophages needs further investigation. Similarly, whether low-dose TSA would change the acetylation levels of both pro-inflammatory and anti-inflammatory targets to skew the activation and polarization of macrophages is worth being studied in the future.

While targeting TAMs has been highlighted as an attractive alternative to classic tumor treatment, the only option to date that has shown promise is cytokine blockade and pan-macrophage depletion [9]. Attempts have been made to pharmacologically reduce TAMs in glioma, for example with CSF-1R inhibitors that decreased M2-like TAMs and increased survival in mouse transgenic and human xenograft glioblastoma models [48]. These strategies have all shown promise, in combination with checkpoint immunotherapies, in preclinical studies that have transitioned into ongoing clinical trials for the treatment of cancers. Unfortunately, CSF-1R inhibitor PLX3397 had no effect in a phase II clinical trial in recurrent glioblastoma [49]. This may also be partly due to (1) compensatory actions by untargeted monocytes, granulocytes, and/or tissue resident macrophages may limit the therapeutic efficacy of such strategies. For instance, targeting granulocytes can lead to the subsequent compensatory expansion of monocytes and macrophages [50], suggesting that the nonselective targeting of all tumor-infiltrating myeloid cells may represent an optimal therapeutic strategy to boost anti-tumor immunity; (2) overall reduction of phagocytic abilities of monocytes/macrophages in tumors, hampering ADCC/ADCP, the major anti-tumor mechanisms of action for many antibody-based therapies. In fact, ADCC, which is mediated by macrophages in addition to NK cells, is an essential component of anti-tumor monoclonal antibody therapies, including those targeting CTLA-4, CD20, and HER2 [13]. In a preclinical lymphoma model, the anti-cancer activity of rituximab was shown to be dependent on chemokine-mediated macrophage recruitment and macrophage effector functions [51]. Moreover, tumor treatment with HDACi has been shown to enhance their susceptibility to NK cell cytotoxicity or DC phagocytosis in vivo [52–54]. More importantly, HDACi could enhance trastuzumab-mediated ADCP and trastuzumab-independent cytotoxicity through upregulating the activating antibody-binding receptor Fc-gamma receptor (FcγR)-II A on monocytes [55]. Given these concerns, the immunostimulatory effect of HDAC inhibition in TAM has compelling advantages over TAM-depleting agents in clinical trials, as it does not reduce macrophage populations in tumors.

## Conclusion

In summary, our findings reveal an immunostimulatory effect of HDAC inhibition that contrasts with those by strategies of depleting or inhibiting TAMs for cancer therapy. Low-dose TSA reduces tumor burden as a single agent by promoting M1-like polarization of TAMs. Our study showing the remarkable efficacy of sequential administration of low-dose TSA and anti-PD-L1 in tumors provides a strong rationale for combination therapy in clinical trials.

## Materials and methods

Further details are provided in the supplemental materials and methods. The primers used for Q-PCR were listed in Supplementary Table 1.

### Animal

C57BL/6, nude mice and BALB/c (both male and female mice, 6–8 weeks of age) were purchased from Shanghai SLAC Laboratory Animal Co. Ltd. (Shanghai, China). RAG2 KO mice were purchased from Jackson Laboratories (Bar Harbor, ME). Mice were housed in cages with a constant-flow air exchange supporting specific pathogen-free conditions. The mice were supplied with irradiated food from Shanghai Pu Lu Teng Biotechnology Co. Ltd. Animal care was in full compliance with the guide for the care and use of laboratory animals and the Institutional Animal Care and Use Committee of the Soochow University approved all protocols. Mice were randomly assigned to experimental groups, and ages and genders were matched.

### Statistical analysis

GraphPad Prism 8.0 (GraphPad Software) was used for statistical analysis. Results are presented as means ± SEM for at least three independent experiments, unless otherwise indicated. Unpaired two-tailed Student’s *t* test were used to compare two groups of independent samples, and one-way or two-way analysis of variance (ANOVA) with Tukey’s post hoc test was used to compare multiple groups. Sample sizes (n) are indicated in the figures or figure legends. Results with *P* values of <0.05 were considered to be statistically significant.

## Supplementary information

Supplementary materials and methods

Supplementary Figure legends

Supplementary Table 1

Supplementary Figure 1

Supplementary Figure 2

Supplementary Figure 3

Supplementary Figure 4

Supplementary Figure 5

Supplementary Figure 6

Supplementary Figure 7

## Data Availability

The authors declare that all relevant data of this study are available within the article or from the corresponding author on reasonable request.

## References

[1] Li X, Song W, Shao C, Shi Y, Han W (2019). Emerging predictors of the response to the blockade of immune checkpoints in cancer therapy. Cell Mol Immunol.

[2] Li X, Shao C, Shi Y, Han W (2018). Lessons learned from the blockade of immune checkpoints in cancer immunotherapy. J Hematol Oncol.

[3] Demaria O, Cornen S, Daeron M, Morel Y, Medzhitov R, Vivier E (2019). Harnessing innate immunity in cancer therapy. Nature.

[4] Coussens LM, Pollard JW (2011). Leukocytes in mammary development and cancer. Cold Spring Harb Perspect Biol.

[5] Pollard JW (2009). Trophic macrophages in development and disease. Nat Rev Immunol.

[6] Medrek C, Ponten F, Jirstrom K, Leandersson K (2012). The presence of tumor associated macrophages in tumor stroma as a prognostic marker for breast cancer patients. BMC Cancer.

[7] Ino Y, Yamazaki-Itoh R, Shimada K, Iwasaki M, Kosuge T, Kanai Y (2013). Immune cell infiltration as an indicator of the immune microenvironment of pancreatic cancer. Br J Cancer.

[8] Li X, Liu R, Su X, Pan Y, Han X, Shao C (2019). Harnessing tumor-associated macrophages as aids for cancer immunotherapy. Mol Cancer.

[9] Mantovani A, Marchesi F, Malesci A, Laghi L, Allavena P (2017). Tumour-associated macrophages as treatment targets in oncology. Nat Rev Clin Oncol.

[10] Noy R, Pollard JW (2014). Tumor-associated macrophages: from mechanisms to therapy. Immunity.

[11] Rodriguez PC, Zea AH, DeSalvo J, Culotta KS, Zabaleta J, Quiceno DG (2003). L-arginine consumption by macrophages modulates the expression of CD3 zeta chain in T lymphocytes. J Immunol.

[12] Gul N, van Egmond M (2015). Antibody-Dependent Phagocytosis of Tumor Cells by Macrophages: a Potent Effector Mechanism of Monoclonal Antibody Therapy of Cancer. Cancer Res.

[13] Romano E, Kusio-Kobialka M, Foukas PG, Baumgaertner P, Meyer C, Ballabeni P (2015). Ipilimumab-dependent cell-mediated cytotoxicity of regulatory T cells ex vivo by nonclassical monocytes in melanoma patients. Proc Natl Acad Sci USA.

[14] Simpson TR, Li F, Montalvo-Ortiz W, Sepulveda MA, Bergerhoff K, Arce F (2013). Fc-dependent depletion of tumor-infiltrating regulatory T cells co-defines the efficacy of anti-CTLA-4 therapy against melanoma. J Exp Med.

[15] Gul N, Babes L, Siegmund K, Korthouwer R, Bogels M, Braster R (2014). Macrophages eliminate circulating tumor cells after monoclonal antibody therapy. J Clin Investig.

[16] Azad N, Zahnow CA, Rudin CM, Baylin SB (2013). The future of epigenetic therapy in solid tumours-lessons from the past. Nat Rev Clin Oncol.

[17] Morel D, Jeffery D, Aspeslagh S, Almouzni G, Postel-Vinay S (2020). Combining epigenetic drugs with other therapies for solid tumours - past lessons and future promise. Nat Rev Clin Oncol.

[18] Topper MJ, Vaz M, Marrone KA, Brahmer JR, Baylin SB (2020). The emerging role of epigenetic therapeutics in immuno-oncology. Nat Rev Clin Oncol.

[19] Loo Yau H, Ettayebi I, De, Carvalho DD (2019). The Cancer Epigenome: exploiting Its Vulnerabilities for Immunotherapy. Trends Cell Biol.

[20] Henning AN, Roychoudhuri R, Restifo NP (2018). Epigenetic control of CD8(+) T cell differentiation. Nat Rev Immunol.

[21] Philip M, Fairchild L, Sun L, Horste EL, Camara S, Shakiba M (2017). Chromatin states define tumour-specific T cell dysfunction and reprogramming. Nature.

[22] Ghoneim HE, Fan Y, Moustaki A, Abdelsamed HA, Dash P, Dogra P (2017). De Novo Epigenetic Programs Inhibit PD-1 Blockade-Mediated T Cell Rejuvenation. Cell.

[23] Amit I, Winter DR, Jung S (2016). The role of the local environment and epigenetics in shaping macrophage identity and their effect on tissue homeostasis. Nat Immunol.

[24] Saeed S, Quintin J, Kerstens HH, Rao NA, Aghajanirefah A, Matarese F (2014). Epigenetic programming of monocyte-to-macrophage differentiation and trained innate immunity. Science.

[25] Minucci S, Pelicci PG (2006). Histone deacetylase inhibitors and the promise of epigenetic (and more) treatments for cancer. Nat Rev Cancer.

[26] Cao K, Wang G, Li W, Zhang L, Wang R, Huang Y (2015). Histone deacetylase inhibitors prevent activation-induced cell death and promote anti-tumor immunity. Oncogene.

[27] Orillion A, Hashimoto A, Damayanti N, Shen L, Adelaiye-Ogala R, Arisa S (2017). Entinostat Neutralizes Myeloid-Derived Suppressor Cells and Enhances the Antitumor Effect of PD-1 Inhibition in Murine Models of Lung and Renal Cell Carcinoma. Clin Cancer Res.

[28] Xie Z, Ikegami T, Ago Y, Okada N, Tachibana M (2020). Valproic acid attenuates CCR2-dependent tumor infiltration of monocytic myeloid-derived suppressor cells, limiting tumor progression. Oncoimmunology.

[29] Franklin RA, Liao W, Sarkar A, Kim MV, Bivona MR, Liu K (2014). The cellular and molecular origin of tumor-associated macrophages. Science.

[30] van Rooijen N, Kors N, ter Hart H, Claassen E (1988). In vitro and in vivo elimination of macrophage tumor cells using liposome-encapsulated dichloromethylene diphosphonate. Virchows Arch B Cell Pathol Incl Mol Pathol.

[31] Tang H, Liang Y, Anders RA, Taube JM, Qiu X, Mulgaonkar A (2018). PD-L1 on host cells is essential for PD-L1 blockade-mediated tumor regression. J Clin Investig.

[32] Lin H, Wei S, Hurt EM, Green MD, Zhao L, Vatan L (2018). Host expression of PD-L1 determines efficacy of PD-L1 pathway blockade-mediated tumor regression. J Clin Investig.

[33] Xiong H, Mittman S, Rodriguez R, Moskalenko M, Pacheco-Sanchez P, Yang Y (2019). Anti-PD-L1 Treatment Results in Functional Remodeling of the Macrophage Compartment. Cancer Res.

[34] Murray PJ, Allen JE, Biswas SK, Fisher EA, Gilroy DW, Goerdt S (2014). Macrophage activation and polarization: nomenclature and experimental guidelines. Immunity.

[35] Klug F, Prakash H, Huber PE, Seibel T, Bender N, Halama N (2013). Low-dose irradiation programs macrophage differentiation to an iNOS(+)/M1 phenotype that orchestrates effective T cell immunotherapy. Cancer Cell.

[36] Nguyen A, Ho L, Workenhe ST, Chen L, Samson J, Walsh SR (2018). HDACi Delivery Reprograms Tumor-Infiltrating Myeloid Cells to Eliminate Antigen-Loss Variants. Cell Rep..

[37] Ugel S, De Sanctis F, Mandruzzato S, Bronte V (2015). Tumor-induced myeloid deviation: when myeloid-derived suppressor cells meet tumor-associated macrophages. J Clin Investig.

[38] Van Dyken SJ, Locksley RM (2013). Interleukin-4- and interleukin-13-mediated alternatively activated macrophages: roles in homeostasis and disease. Annu Rev Immunol.

[39] Pucci F, Venneri MA, Biziato D, Nonis A, Moi D, Sica A (2009). A distinguishing gene signature shared by tumor-infiltrating Tie2-expressing monocytes, blood “resident” monocytes, and embryonic macrophages suggests common functions and developmental relationships. Blood.

[40] Guerriero JL, Sotayo A, Ponichtera HE, Castrillon JA, Pourzia AL, Schad S (2017). Class IIa HDAC inhibition reduces breast tumours and metastases through anti-tumour macrophages. Nature.

[41] Marvel D, Gabrilovich DI (2015). Myeloid-derived suppressor cells in the tumor microenvironment: expect the unexpected. J Clin Investig.

[42] Flores-Toro JA, Luo D, Gopinath A, Sarkisian MR, Campbell JJ, Charo IF (2020). CCR2 inhibition reduces tumor myeloid cells and unmasks a checkpoint inhibitor effect to slow progression of resistant murine gliomas. Proc Natl Acad Sci USA.

[43] Lu Z, Zou J, Li S, Topper MJ, Tao Y, Zhang H (2020). Epigenetic therapy inhibits metastases by disrupting premetastatic niches. Nature.

[44] Sun SC (2017). The non-canonical NF-kappaB pathway in immunity and inflammation. Nat Rev Immunol.

[45] Kim YD, Park SM, Ha HC, Lee AR, Won H, Cha H (2020). HDAC Inhibitor, CG-745, Enhances the Anti-Cancer Effect of Anti-PD-1 Immune Checkpoint Inhibitor by Modulation of the Immune Microenvironment. J Cancer.

[46] Halili MA, Andrews MR, Labzin LI, Schroder K, Matthias G, Cao C (2010). Differential effects of selective HDAC inhibitors on macrophage inflammatory responses to the Toll-like receptor 4 agonist LPS. J Leukoc Biol.

[47] Cui SN, Chen ZY, Yang XB, Chen L, Yang YY, Pan SW (2019). Trichostatin A modulates the macrophage phenotype by enhancing autophagy to reduce inflammation during polymicrobial sepsis. Int Immunopharmacol.

[48] Pyonteck SM, Akkari L, Schuhmacher AJ, Bowman RL, Sevenich L, Quail DF (2013). CSF-1R inhibition alters macrophage polarization and blocks glioma progression. Nat Med.

[49] Butowski N, Colman H, De Groot JF, Omuro AM, Nayak L, Wen PY (2016). Orally administered colony stimulating factor 1 receptor inhibitor PLX3397 in recurrent glioblastoma: an Ivy Foundation Early Phase Clinical Trials Consortium phase II study. Neuro Oncol.

[50] Nywening TM, Belt BA, Cullinan DR, Panni RZ, Han BJ, Sanford DE (2018). Targeting both tumour-associated CXCR2(+) neutrophils and CCR2(+) macrophages disrupts myeloid recruitment and improves chemotherapeutic responses in pancreatic ductal adenocarcinoma. Gut.

[51] Cittera E, Leidi M, Buracchi C, Pasqualini F, Sozzani S, Vecchi A (2007). The CCL3 family of chemokines and innate immunity cooperate in vivo in the eradication of an established lymphoma xenograft by rituximab. J Immunol.

[52] Christiansen AJ, West A, Banks KM, Haynes NM, Teng MW, Smyth MJ (2011). Eradication of solid tumors using histone deacetylase inhibitors combined with immune-stimulating antibodies. Proc Natl Acad Sci USA.

[53] Armeanu S, Bitzer M, Lauer UM, Venturelli S, Pathil A, Krusch M (2005). Natural killer cell-mediated lysis of hepatoma cells via specific induction of NKG2D ligands by the histone deacetylase inhibitor sodium valproate. Cancer Res.

[54] Skov S, Pedersen MT, Andresen L, Straten PT, Woetmann A, Odum N (2005). Cancer cells become susceptible to natural killer cell killing after exposure to histone deacetylase inhibitors due to glycogen synthase kinase-3-dependent expression of MHC class I-related chain A and B. Cancer Res.

[55] Laengle J, Kabiljo J, Hunter L, Homola J, Prodinger S, Egger G (2020). Histone deacetylase inhibitors valproic acid and vorinostat enhance trastuzumab-mediated antibody-dependent cell-mediated phagocytosis. J Immunother Cancer.

