# Supplementary material for: HDAC inhibition potentiates anti-tumor activity of macrophages and enhances anti-PD-L1-mediated tumor suppression
Source: Oncogene. 2021 Feb 9;40(10):1836–50. doi: 10.1038/s41388-020-01636-x (PMC7946638; doi:10.1038/s41388-020-01636-x)
Supplement: Supplementary file 1 — Supplementary materials and methods [file 41388_2020_1636_MOESM1_ESM.docx]

**Supplementary Materials and Methods**

**Cell lines and Reagents.** B16F10 (melanoma), 4T1 (breast cancer), and LLC (Lewis lung carcinoma) were obtained from the American Type Culture Collection (ATCC). All cell lines were examined and authenticated by short tandem repeat profiling. All cell lines were Mycoplasma negative and used within 10 passages. Cell lines were cultured in DMEM supplemented with 10% FBS, 100 IU/ml penicillin, and 100 IU/ml streptomycin. Prior to use, cells with 70%-80% of confluence were detached with trypsin-EDTA 0.25% and washed in PBS for two times. To collect cell supernatants, cells were seeded in 6-well plates at a density of 1×10^6^ cells/ml in complete DMEM medium. The cell supernatant was collected 24 h later, filtered through a 0.22 μm filter and stored at − 80 °C. InVivo Mab anti-mouse PD-L1(B7-H1) antibody (clone 10F.9G2) was purchased from BioXCell and was administered intraperitoneally. Two HDAC inhibitors trichostatin-A (TSA) and vorinistat (SAHA) were purchased from MedChem Express, MCE. Clodronate liposomes were purchased from [www.clodronateliposomes.org](http://www.clodronateliposomes.org). The following antibodies were used for flow cytometry analysis of tumors, spleen, blood and tumor-draining lymph nodes. Anti-CD45 (clone 30-F11), anti-CD3 (clone 17A2), anti-CD4 (clone GK1.5), anti-CD8 (clone 53-6.7), anti-CD11b (clone M1/70), anti-F4/80 (clone BM8), anti-I-A/I-E (clone M5/114/15.2), anti-Gr1 (clone RB6-8C5), anti-CD206 (clone C068C2), anti-Arg1 (clone A1exF5), anti-Nos2 (clone CXFNT) were purchased from eBioscience. Anti-TNF-a (clone MP6-XT22), anti-granzyme B (clone GB11), anti-IFN-g (clone XMG1.2), anti-Ki67 (clone B56), anti-Foxp3 (clone MF-14) were purchased from BioLegend.

**Tumor models.** All animal experiments were in full compliance with the guide for the care and use of laboratory animals and the Institutional Animal Care and Use Committee of the Soochow University approved all protocols. B16F10 and LLC tumors were generated by subcutaneous (s.c.) injection of 5 × 10^5^ in 6-wk-old C57BL/6 mice. 4T1 tumor (5 × 10^5^) were implanted s.c. into the skin of 6-wk-old BALB/c mice. Tumors were grown for several weeks, according to either a survival schedule (endpoint defined by tumor volume) or a fixed time point. Tumor size was measured by calipers, and the volume was calculated according to the formula (*D* ×*d* × *d*)/2, with *D* and *d* being the longest and shortest tumor diameter, respectively, in millimeters. Animals were killed at experimental termination or when predetermined Institutional Animal Care and Use Committee rodent health endpoints were reached.

**Treatment of tumor-bearing mice with TSA and anti-PD-L1.** TSA was dissolved in dimethyl sulphoxide (DMSO) and diluted in phosphate-buffered saline (PBS). For the TSA treatment protocol, TSA (0.5 mM/kg, daily) was injected intraperitoneally (i.p.) after successful establishment of the tumors (at least 50 to 120 mm^3^). For the anti-PD-L1 treatment, mice were inoculated s.c. injection with tumor cells (5 × 10^5^/mice). On day 8 after palpable tumors formed, mice were treated with low-dose HDACi TSA (0.5 mM/kg, i.p. injection, daily) in combination with or without anti-PD-L1 (200 mg/mouse, i.p. injection, 3 times).

**Isolation of single cells from mouse tumors.** Tumors were isolated, minced in a petri dish on ice and then enzymatically dissociated in Hanks balanced salt solution containing collagenase I (0.5 mg/ml, Gibco), collagenase IV (1 mg/ml, Gibco), hyaluronidase (1 mg/ml, Sigma) and DNase I (20 U/ml, Sigma) at 37 °C for 30 min. Cell suspensions were filtered through a 70-mm cell strainer. Briefly, cells were pelleted, resuspended in 70% percoll (GE healthcare, Piscataway, NJ, USA), overlaid with 40% percoll, and then centrifuged at 2000 r.p.m. for 20 min at room temperature. The resulting suspension was filtered through a cell strainer to produce a single-cell suspension. Cells were washed once with PBS before use in flow cytometry analysis.

**Flow cytometry staining and analysis.** Single-cell suspensions from tumors, spleen, blood and tumor-draining lymph nodes were stained, using a Live/Dead Fixable Aqua Dead Cell Stain Kit (Life Technologies), for dead cell exclusion according to the manufacturer’s instructions, in combination with anti-mouse CD16/CD32 FcR-blocking reagent (BD Biosciences) for 15 min on ice in the dark. Cells were washed and incubated with fluorochrome-conjugated antibodies directed against cell surface antigens at the manufacturer’s recommended dilution for 30 min on ice in the dark, washed, and resuspended in fluorescence-activated cell sorter (FACS) buffer (PBS + 2% FBS). For IFN-γ and TNF-α staining, cells were stimulated in vitro with a cell stimulation cocktail (plus protein transport inhibitors, eBioscience) for 4-6 hours. Cells were then processed using a fixation and permeabilization kit (BD Bioscience) and stained with IFN-γ and TNF-α antibodies which were purchased from eBioscience. For intracellular staining, cells stained with cell surface antibodies were fixed, permeabilized using transcription factor staining buffer set prior to incubation with antibodies directed at intracellular antigens (eBioscience). Multicolour FACS analysis was performed on a BD Canto RUO 11 colour analyser. All data analysis was performed using the flow cytometry analysis program FlowJo (Treestar, OR, USA).

**Clodronate treatment (macrophages depletion).** Clodronate was encapsulated in liposomes to create clodronate liposomes. Control liposomes contained PBS. When the average tumor size was 150 mm^3^, mice were treated i.p. injection with 200 μl clodronate-encapsulated liposomes per mouse every 3 days for 2 weeks in combination with daily administration of vehicle or low-dose TSA.

**TAMs isolation.** Tumors were isolated, minced in a petri dish on ice and then enzymatically dissociated in Hanks balanced salt solution containing collagenase I (0.5 mg/ml, Gibco), collagenase IV (1 mg/ml, Gibco), hyaluronidase (1 mg/ml, Sigma) and DNase I (20 U/ml, Sigma) at 37 °C for 30 min. The lymphomononuclear correspondent layer was isolated and cultured for 40 min at 37 °C. After three washes, the adherent cells remained in the plate. Prior to use, adherent cells were removed using a cell scraper (1, 2). TAMs (CD11b^+^F4/80^+^) were 70% to 80% pure, as confirmed through flow cytometry.

**In vivo macrophages adoptive transfer experiments.** LLC cells (5 × 10^5^ in 100 μl PBS) were injected subcutaneously into the flanks of mice at day 0. When the average tumor size was 150 mm^3^, mice were treated by intraperitoneally injection with DMSO or TSA every day. On day 21 post tumor challenge, mice were sacrificed. TAMs from tumor-bearing mice treated with DMSO or TSA were enriched. Then, LLC cells (5 × 10^5^) were subcutaneously co-injected with or without TAMs isolated from DMSO or TSA treatment groups (5 × 10^5^) into the flanks of new host mice. Tumor dimensions were measured three times per week beginning on day 7.

**L929-differentiation medium and tumor-conditioned media.** L929 cells were acquired from the American Type Culture Collection (ATCC) and cultured in the DMEM (Gibco, MA, USA) supplied with 2 mM L-glutamine, 1.0 mM sodium pyruvate (all from Gibco, MA, USA), and 10% fetal bovine serum (FBS, Hyclone, MA, USA). Cells were maintained in a humidified 37 °C CO2 incubator and passed at 70% confluence. To generate conditioned medium, media was removed from 70% confluent cultures and replaced with fresh media. After 6 days, culture supernatants were collected and aliquots stored at − 80 °C. LLC, B16, 4T1 cancer cells were cultured in DMEM supplied with 10% FBS. Cells were maintained in a humidified 37 °C CO2 incubator and passed at 70% confluence. To generate conditioned medium, media was removed from 70% confluent cultures and replaced with fresh media. After 3 days, culture supernatants were collected and aliquots stored at − 80 °C.

**Mouse macrophage differentiation and culture.** To generate bone marrow-derived macrophages, mice were sacrificed and disinfected with 70% ethanol. Both lower extremities were excised, and the long bones (femur and tibia) from healthy male C57BL/6 was separated from muscular layers and placed in DMEM/F12 medium (Gibco, MA, USA). To extract bone marrow-derived macrophages, 10 ml DMEM/F12 medium was used to flush out each bone using a 25-gauge needle, and cells were gently dissociated by pipetting. A 70-μm nylon BD Falcon cell strainer was placed atop a 50-ml BD Falcon tube, and the suspension was filtered into the 50-ml tube. The resultant suspension was centrifuged at 1500 rpm for 5 min. The cells were washed 2 additional times with 1 × PBS. The cells were resuspended in DMEM/F12 medium containing 10% FBS (v/v, Hyclone, MA, USA) and 20% L929 conditioned medium (v/v), seeded in ultra-low attachment culture dishes and cultured at 37 °C with 5% CO2 in a humidified atmosphere for 6 days. Fresh differentiation medium was added on day 4. TSA was added on days 1 and 4. These cells were maintained in a standard 37 °C with 5% CO2 incubator.

**Macrophage polarization.** Bone-marrow-derived macrophages were isolated and cultured in DMEM/F12 medium containing 10% FBS (v/v), penicillin (100 U/ml), amphotericin B (2 μg/ml), and 20% of L929 cell culture supernatant (v/v) for 7 days, at 37 °C in a 5% CO2 atmosphere. Fresh differentiation medium was added on day 4. TSA was added on days 1 and 4. Bone-marrow-derived macrophages were polarized with IFN-γ (20 ng/ml, Peprotech) + LPS (100 ng/ml, Sigma) or LPS alone for 24 h, or IL-4 (20 ng/ml, Peprotech) + IL-13 (20 ng/ml, Peprotech) for 24 h. Total RNA was harvested from macrophages using the RNeasy Mini Kit according to the manufacturer’s instructions.

**Measurement of NO.** Macrophages were plated in flat-bottom 96-well plates at 106/ml and supernatant was collected at 24 h and 48 h for NO. Nitrite (NO^2-^) quantification was assayed by a standard Greiss reaction. Culture supernatant (100 ml) was mixed with 100 ml of 1% sulfanilamide, 0.1% N-(1-naphthy)-ethylenediamine dihydrochloride, and 2.5% H_3_PO_4_. Absorbance was measured at 540 nm in a microplate reader.

**Co-culture of macrophages and T cells.** This experiment was conducted as described previously (3). Briefly, T cells were enriched from the spleen of C57BL6/J mice using a Pan T Cell Isolation Kit. Then, isolated T cells were re-suspended in 1 ml of PBS with 2.5 μM CSFE (CellTrace™ CSFE Cell Proliferation Kit, Invitrogen) and incubated at 37 °C in a 5% CO2 atmosphere for 20 min. In all, 35 ml of RMPI with 10% FBS were subsequently added to the cells and incubated for an additional 10 min. Subsequently, T cells were washed twice in PBS with 2% FBS. CSFE-labeled T cells (10^5^) were cultured in a 96-well plate, pre-coated with anti-CD3, and soluble anti-CD28 was added to the medium to induce T-cell proliferation either with or without macrophages at the indicated ratios. Three days later, cells were collected and analyzed by flow cytometry.

**Immunohistochemistry.** IHC was performed on the Leica Bond automated staining platform. Tumors were extracted from tumor-bearing mice and fixed in 10% formalin overnight after which they were embedded in paraffin and sectioned. Prior to immunohistochemical staining, tumor sections were washed twice with Histo-Clear II, followed by two washes with 100% ethanol, and subsequent hydration with washes of 90%, 80%, 70%, and 50% ethanol. Tumor sections were heated in 10 mM sodium citrate buffer (pH 6.0) for antigen unmasking. After cooling, sections were washed in de-ionized water, then incubated in 3% Hydrogen peroxide for 10 min at room temperature followed by washes in de-ionized water and 1× PBS. For antigen blocking, sections were incubated in PBS buffer containing 0.5% Tween, 1% BSA plus 5% serum for 1 h at room temperature. Sections were then stained in block buffer containing primary antibodies: CD4, CD8, Ki67, Nos2, and Arg1 overnight in a wet chamber at 4°C in the dark. CD4, CD8, Ki67, Nos2, and Arg1 antibodies were purchased from Abcam. The following day, sections were washed three times in 1×PBS and then stained with secondary biotinylated antibody in PBS blocking buffer for 1 h at room temperature. Sections were washed three times with 1×PBS and Elite Vectastain ABC Kit was applied for 30 minutes following the manufacturer’s instructions at room temperature in the dark. Sections were washed with 1×PBS, developed with DAB reagent, and counterstained with hematoxylin. Sections were then washed twice with de-ionized water followed by one wash with 1×PBS, and additional washes of increasing ethanol concentration for dehydration followed by incubation in Histo-Clear II. Slides were mounted with Vecta-Mount Permanent Mounting Medium and covered with glass coverslips. After 24 hours, sections were viewed with an Olympus BX43 Trinocular Microscope.

**H&E staining.** Tumor tissues were collected and fixed in 4% paraformaldehyde overnight. Tumors were then dehydrated through sequentially treatment with 75% ethanol (1 h), 95% ethanol (1 h for twice) and 100% ethanol (1 h for twice). The samples were treated with xylene for 20 min twice before embedded in paraffin. The samples were then sectioned. Histology was performed using standard Hematoxylin & Eosin (H&E). Micrographs were captured using a Zeiss Observer Z1 (Carl Zeiss, Jena, Germany).

**Immunofluorescence.** Tumors were excised from tumor-bearing mice, embedded in and immediately snap-frozen in O.C.T. compound (Fisher Healthcare). Frozen fresh tissues were cryo-sectioned and sections were fixed for 10 minutes in pre-cooled (−20°C) acetone, washed three times in ice cold 1×PBS, and incubated for 30 min in blocking buffer (10% goat or rabbit serum in PBS). Slides were then incubated with primary antibody diluted in 2% serum in PBS in a humidity chamber overnight at 4°C. After washing 3 times with 2% serum diluted in PBS each for 5 min, slides were incubated with secondary antibody diluted in 2% serum at room temperature for 2 h. Nuclei were stained with 100 ng/ml Hoechst in PBS and incubated for 10 min at room temperature in the dark. Slides were washed twice with PBS, and imaged with Leica confocal microscope. Sequential acquisitions of the multicolor images were used to avoid cross-excitation, and images were overlaid with Leica Confocal Microscope Imaging Software (Leica).

**RNA extraction, RT-PCR and qPCR.** Total RNA was extracted from sorted mouse macrophages and TAMs using Trizol reagent or using the RNeasy Mini Kit according to the manufacturer’s recommendations (Invitrogen). Genomic DNA contamination was eliminated by treatment with DNase I (Life Technologies). Reverse transcription was performed with the Prime Script RT Master Mix (Takara, RR036A). RNA samples (0.5-1 μg) were converted to first-strand cDNAs using random and oligo-(dT)15 primer mixture (1:1). The cDNA samples were diluted 1:10 in water and the FastStart Universal SYBR Green Master Mix (Roche, 04913914001) was added along with cDNA and 1 pmol primers for a total PCR reaction volume of 10 μl. Primer sequences were summarized in Supplementary Table 1. Relative gene expression level of each respective genes was calculated using the threshold cycle method and normalized to β-actin.

**Western blotting.** Western blot was performed as described previously (4). Briefly, bone marrow-derived macrophages were washed three times with 2 ml of ice-cold PBS. Cells were lysed in 1% Triton X-100 in TBS, pH 7.6, with Roche complete protease inhibitor for 30 min on ice followed by pelleting of insoluble material by centrifugation. The lysates concentration of each sample was determined by Protein Assay (Bio-Rad, M60-009RDPD). Lysates were heated on 95 °C in SDS sample buffer with 50 mM DTT for 10 min. Thirty μg proteins were loaded and separated on SDS-PAGE gels. Proteins were transferred onto PVDF membrane by wet transfer. PVDF membranes were blocked in TBST buffer containing 5% defatted milk powder for 2 h prior to probing with primary antibodies. Images were acquired with HRP-conjugated secondary antibodies. All antibodies were used according to the manufacturer’s instructions.

**Statistical analysis.** Results are expressed as mean ± standard error of the mean (S.E.M.). All data analysis was performed using two-tailed unpaired Student’s t-test or other statistical methods indicated in the text with GraphPad Prism software (version 8.0, GraphPad Software, Inc.). For each parameter of all data * indicates *P*<0.05, ** indicates *P*<0.01, *** indicates *P*<0.001 and *****P* < 0.0001, ns, no significant.

**References**

1. J. M. Mota et al., Post-Sepsis State Induces Tumor-Associated Macrophage Accumulation through CXCR4/CXCL12 and Favors Tumor Progression in Mice. Cancer Immunol Res 4, 312-322 (2016).

2. C. W. Wanderley et al., Paclitaxel Reduces Tumor Growth by Reprogramming Tumor-Associated Macrophages to an M1 Profile in a TLR4-Dependent Manner. Cancer Res 78, 5891-5900 (2018).

3. L. Du et al., IGF-2 Preprograms Maturing Macrophages to Acquire Oxidative Phosphorylation-Dependent Anti-inflammatory Properties. Cell Metab 29, 1363-1375 e1368 (2019).

4. X. Li et al., Blockade of the LRP16-PKR-NF-kappaB signaling axis sensitizes colorectal carcinoma cells to DNA-damaging cytotoxic therapy. Elife 6 (2017).
