# Supplementary material for: HDAC inhibition potentiates anti-tumor activity of macrophages and enhances anti-PD-L1-mediated tumor suppression
Source: Oncogene. 2021 Feb 9;40(10):1836–50. doi: 10.1038/s41388-020-01636-x (PMC7946638; doi:10.1038/s41388-020-01636-x)
Supplement: Supplementary file 2 — Supplementary Figure legends [file 41388_2020_1636_MOESM2_ESM.docx]

**Supplementary Figures legends**

**Supplementary Fig. 1. Specific HDAC overexpression is positively correlated with poor prognosis in patients with either breast cancer (a) or lung cancer (b).** Data was obtained from an online database of potential cancer biomarkers (http://www.kmplot.com) to assess the prognostic implications of HDAC1, HDAC2, HDAC4, and HDAC10 expression in breast cancer patients and of HDAC1, HDAC2, HDAC4, HDAC10, and HDAC11 expression in lung cancer patients.

**Supplementary Fig. 2. Administration of low-dose TSA inhibits tumor growth through the intact immune system. (a)** B16F10 cells were injected s.c. in the flank of C57BL/6 mice. Starting on day 8 after tumor inoculation, we treated tumor-bearing mice i.p. with 0.5 μM/kg or 3 μM/kg TSA, with DMSO used as a control daily. Tumor size was measured and plotted as average total tumor volume ± s.e.m. On day 14, tumor weight was measured. Representative H&E-stained sections from B16F10 tumors collected on day 15, (scale bar, 1 mm). **(b)** 4T1 cells were injected s.c. in the flank of BALB/c mice. Starting on day 8 after tumor inoculation, we treated tumor-bearing mice i.p. with 0.5 μM/kg or 3 μM/kg TSA, with DMSO used as a control daily. Tumor size was measured and plotted as average total tumor volume ± s.e.m. **(c)** 4T1 cells were injected s.c. in the flank of BALB/c mice. Tumor size and tumor weight were measured at the indicated day. Representative images of tumors from nude-bearing mice treated with 0.5 μM/kg or 3 μM/kg TSA, with DMSO used as a control. Data are representative of at least three independent experiments. **P* < 0.05, ***P* < 0.01, ns, no significant.

**Supplementary Fig. 3. TSA induces tumor cell death dose-dependently. (a)** melanoma (B16F10), breast cancer (4T1) and lung cancer (LLC) were treated with the indicated concentrations of TSA ex vivo for 48 h, and found higher dosage TSA do reduce cell viability and also induce tumor cell death **(b)**. Data are representative of at least three independent experiments.

**Supplementary Fig. 4. Addition of low-dose TSA could not significantly alter systemic T cell content. (a)** 4T1 cells were injected s.c. in the flank of BALB/c mice. Tumor-bearing mice were treated i.p. with 0.5 μM/kg or DMSO used as a control. Percentages of CD45^+^ cells, CD3^+^ T cells, CD4^+^ and CD8^+^ T cells in blood and spleen were determined by FACS. **(b)** B16F10 cells were injected s.c. in the flank of C57BL/J mice. Tumor-bearing mice were treated i.p. with 0.5 μM/kg or DMSO used as a control. Percentages of CD45^+^ cells, CD3^+^ T cells, CD4^+^ and CD8^+^ T cells within tumors were determined by FACS. **(c)** Tregs cells (Foxp3^+^ cells) in TILs, blood and spleen were determined by FACS. **(d)** Ki-67 expression in CD4^+^ and CD8^+^ T cells obtained from blood, spleen and TILs was determined by intracellular cytokine staining. **(e)** T cells were treated with the indicated concentrations of TSA ex vivo for 72 h. T cell proliferation assay was performed. **(f)** Production of IFN-γ and TNF-α by CD4^+^ and CD8^+^ T cells within tumor-draining lymph node was determined by intracellular cytokine staining. Data are representative of at least three independent experiments. **P*< 0.05, ns, no significant.

**Supplementary Fig. 5. HDAC inhibition can also inhibit the trafficking of MDSCs into tumors. (a)** 4T1 tumor-bearing mice were received daily i.p. injections of either vehicle (DMSO) or low-dose TSA. Percentage of MDSCs within tumors was determined by FACS. **(b)** B16F10 tumor-bearing mice were received daily i.p. injections of either vehicle (DMSO) or low-dose TSA. Percentage of MDSCs within tumors was determined by FACS. **(c)** The percentages of TAMs and the absolute TAMs numbers within tumors of WT mice treated with DMSO control or low-dose TSA. **(d)** Gating strategy for MHC-II^+^CD11b^low^ versus MHC-II^+^CD11b^hi^. **(e)** T cells from macrophages and T co-culture system were subjected to suppress the activation of CSFE-labeled T cells. **(f)** Percentage of intratumoral CD11b^+^ myeloid population in mice within tumors was determined by FACS. Data are representative of at least three independent experiments. **P* < 0.05, ***P* < 0.01.

**Supplementary Fig. 6. Addition of low-dose TSA could also re-educate mature macrophages ex vivo. (a)** Both maturing and mature macrophages were treated with the indicated concentrations of TSA ex vivo for 48 h, cell viability was detected by CCK-8. **(b)** Mature macrophages from IFN-γ (20 ng/ml) + LPS (100 ng/ml) in the presence or absence of TSA were assayed for the specific gene mRNA expression. **(c)** Mature macrophages were treated with the indicated cytokines to detect the expression level of Nos2. **(d)** Mature macrophages from IL-4 (20 ng/ml) + IL-13 (20 ng/ml) in the presence or absence of TSA (10 nM) were assayed for the specific gene mRNA expression. **(e)** Mature macrophages were treated with the indicated cytokines to detect the expression level of CD206. **(f)** Macrophages from IL-4 (20 ng/ml) + IL-13 (20 ng/ml) or from IFN-γ (20 ng/ml) + LPS (100 ng/ml) in the presence or absence of SAHA (20 nM) were assayed for the specific gene mRNA expression. Mature macrophages from IFN-γ (20 ng/ml) + LPS (100 ng/ml) in the presence or absence of SAHA (20 nM) to detect the expression level of Nos2 **(g)** or from IL-4 (20 ng/ml) + IL-13 (20 ng/ml) in the presence or absence of SAHA (20 nM) to detect the expression level of Cd206 **(h)**. Immunostaining of CD206 **(i)**, Nos2 **(j)** and Arg1**(k)** in macrophages treated with IFN-γ (20 ng/ml) + LPS (100 ng/ml) in the presence or absence of TSA or from IL-4 (20 ng/ml) + IL-13 (20 ng/ml) in the presence or absence of TSA. **(l)** Phenotype of macrophages in the presence of regular medium (RM) or tumor-conditioned medium (TCM). Data are representative of at least three independent experiments. **P* < 0.05, ***P* < 0.01, ****P* < 0.001, *****P* < 0.0001, ns, no significant.

**Supplementary Fig. 7. HDAC inhibition synergizes with anti-PD-L1 to suppress tumor growth. (a-c)** melanoma (B16F10), breast cancer (4T1) and lung cancer (LLC) were treated with the indicated concentrations of TSA ex vivo for 48 h, PD-L1 expression was determined by FACS. (**d** and **e**) the protein and mRNA expression level of PD-L1 in tumor cells and CD11b+ cells were determined by FACS (**d**) and qPCR (**e**). **(f)** tumor-bearing mice were received daily i.p. injections of either vehicle, TSA alone, anti-PD-L1, or anti-PD-L1+ TSA, and tumor growth was measured. **(g)** tumor-bearing mice based on tail vein injection of tumor cells were also treated with either vehicle, TSA alone, anti-PD-L1, or anti-PD-L1 + TSA, and representative images of lungs from tumor-bearing mice. **(h)** Representative IHC images of tumors from mice treated with either vehicle, TSA alone, anti-PD-L1, or anti-PD-L1 + TSA. Data are representative of at least three independent experiments.
