# Supplementary figures and images for: HDAC inhibition potentiates anti-tumor activity of macrophages and enhances anti-PD-L1-mediated tumor suppression
Source: Oncogene. 2021 Feb 9;40(10):1836–50. doi: 10.1038/s41388-020-01636-x (PMC7946638; doi:10.1038/s41388-020-01636-x)

**Supplementary Fig. 1**

**
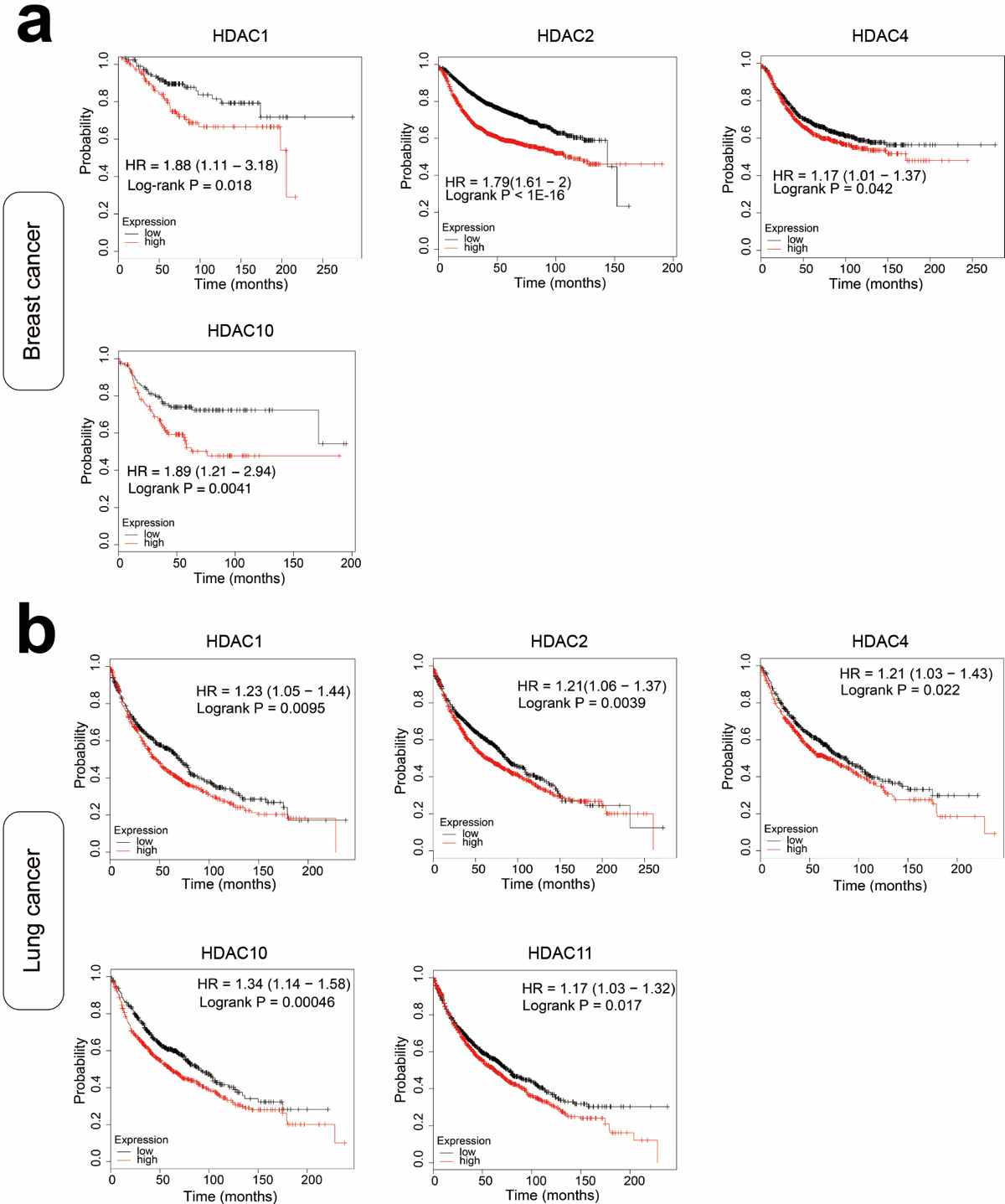
**

Supplement: Supplementary file 4 — Supplementary Figure 1 [file 41388_2020_1636_MOESM4_ESM.docx]

**Supplementary Fig. 2
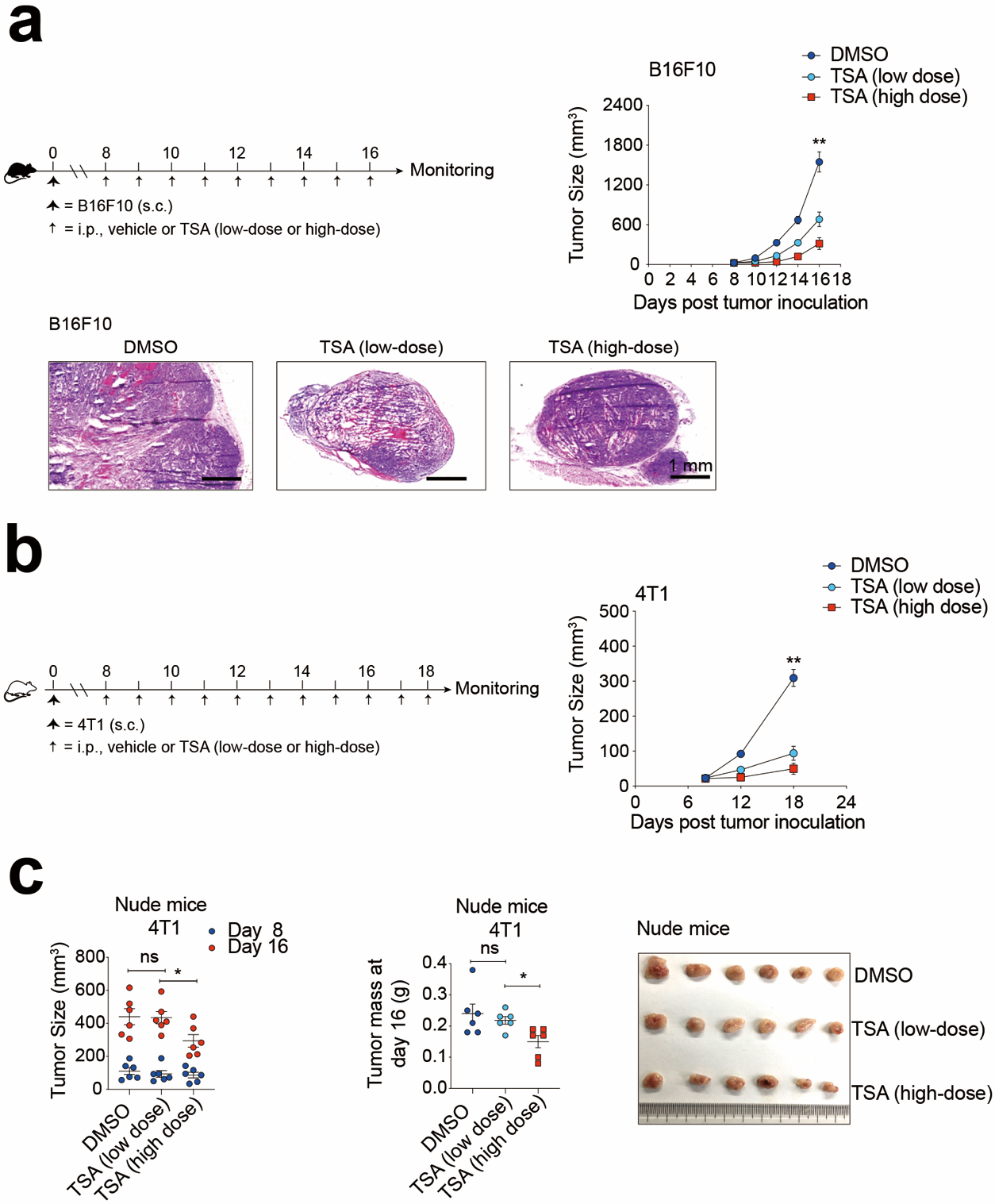
**

Supplement: Supplementary file 5 — Supplementary Figure 2 [file 41388_2020_1636_MOESM5_ESM.docx]

**Supplementary Fig. 3
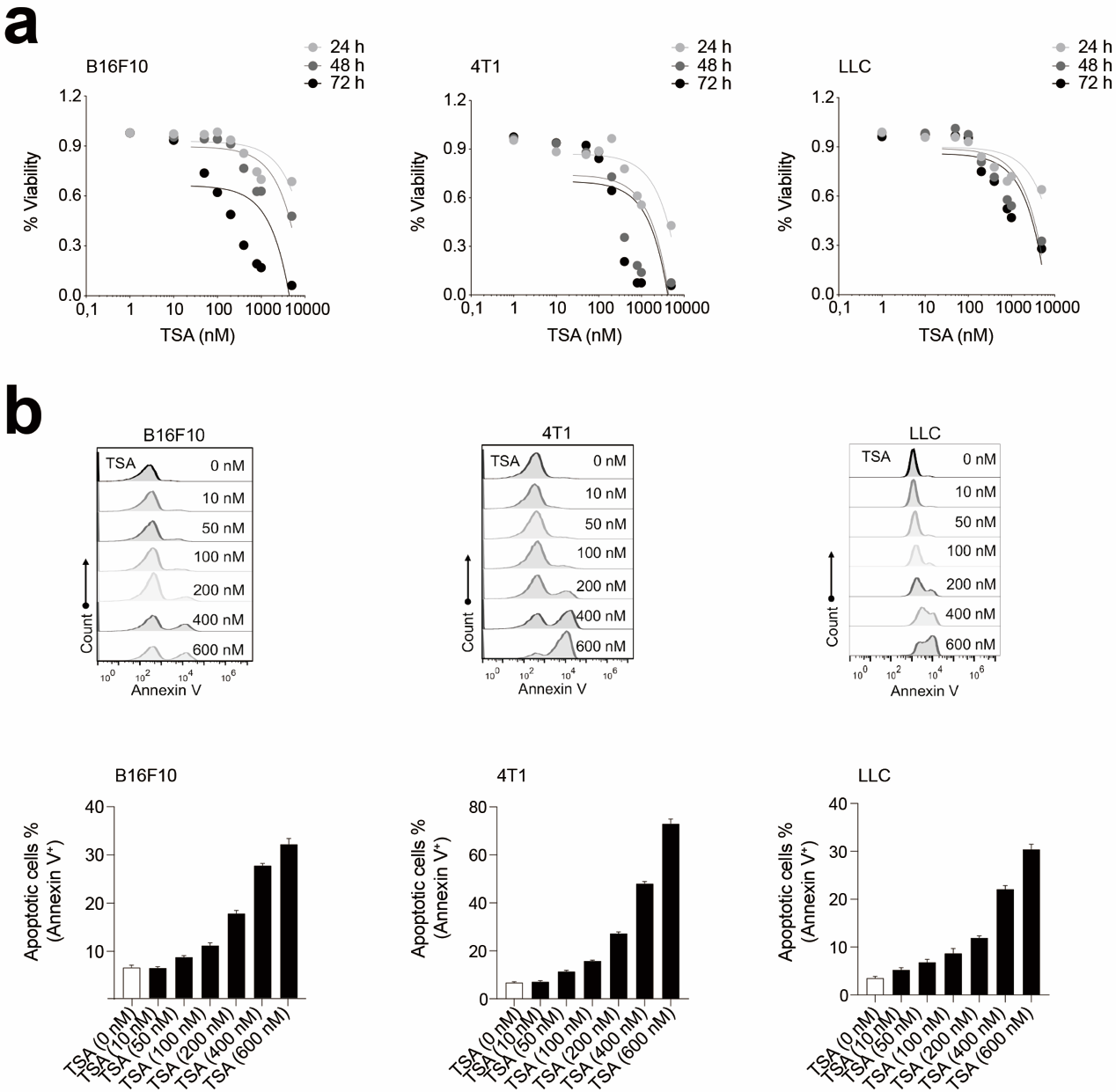
**

Supplement: Supplementary file 6 — Supplementary Figure 3 [file 41388_2020_1636_MOESM6_ESM.docx]

**Supplementary Fig. 4
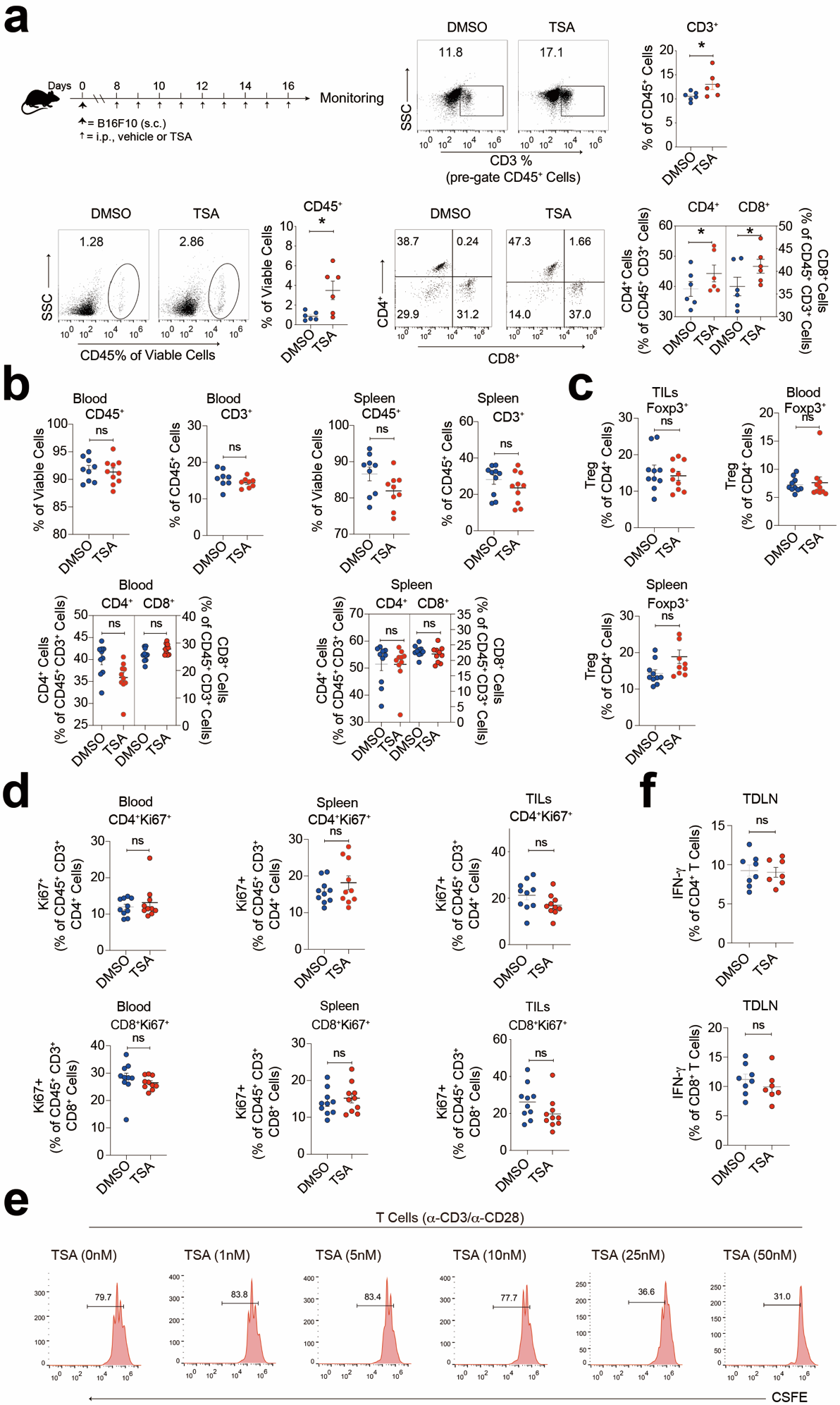
**

Supplement: Supplementary file 7 — Supplementary Figure 4 [file 41388_2020_1636_MOESM7_ESM.docx]

**Supplementary Fig. 5
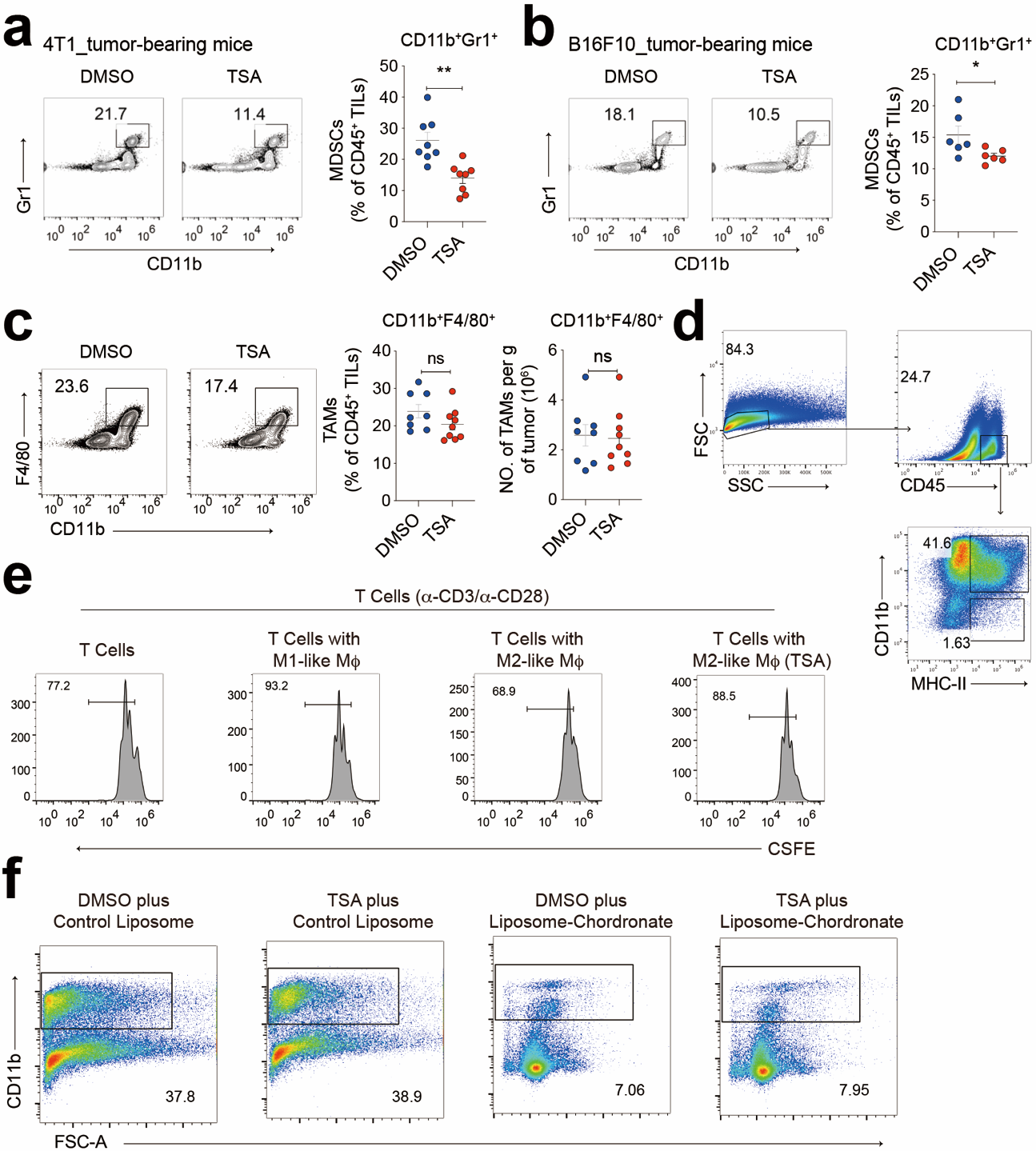
**

Supplement: Supplementary file 8 — Supplementary Figure 5 [file 41388_2020_1636_MOESM8_ESM.docx]

**Supplementary Fig. 6
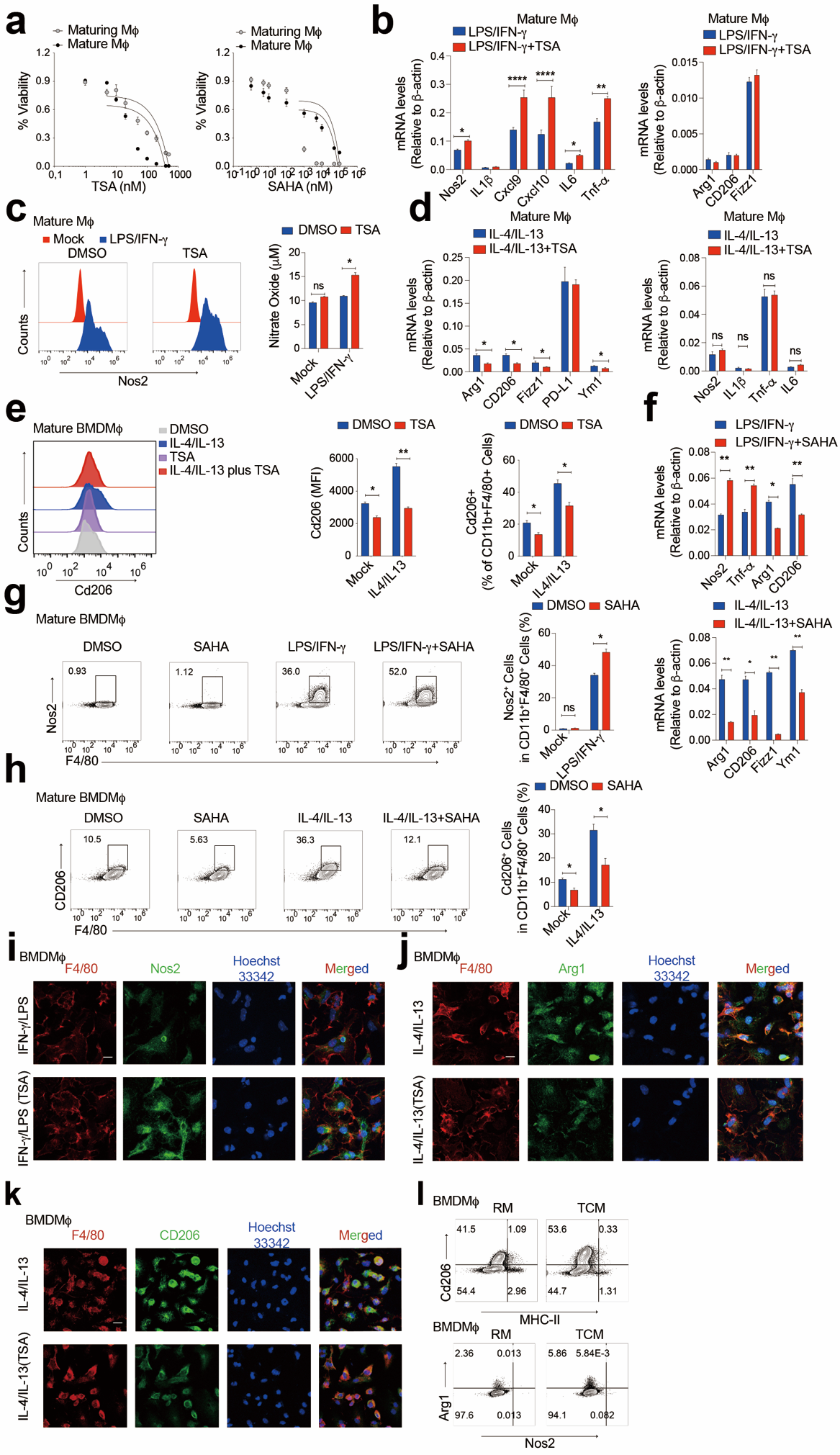
**

Supplement: Supplementary file 9 — Supplementary Figure 6 [file 41388_2020_1636_MOESM9_ESM.docx]

**Supplementary Fig. 7
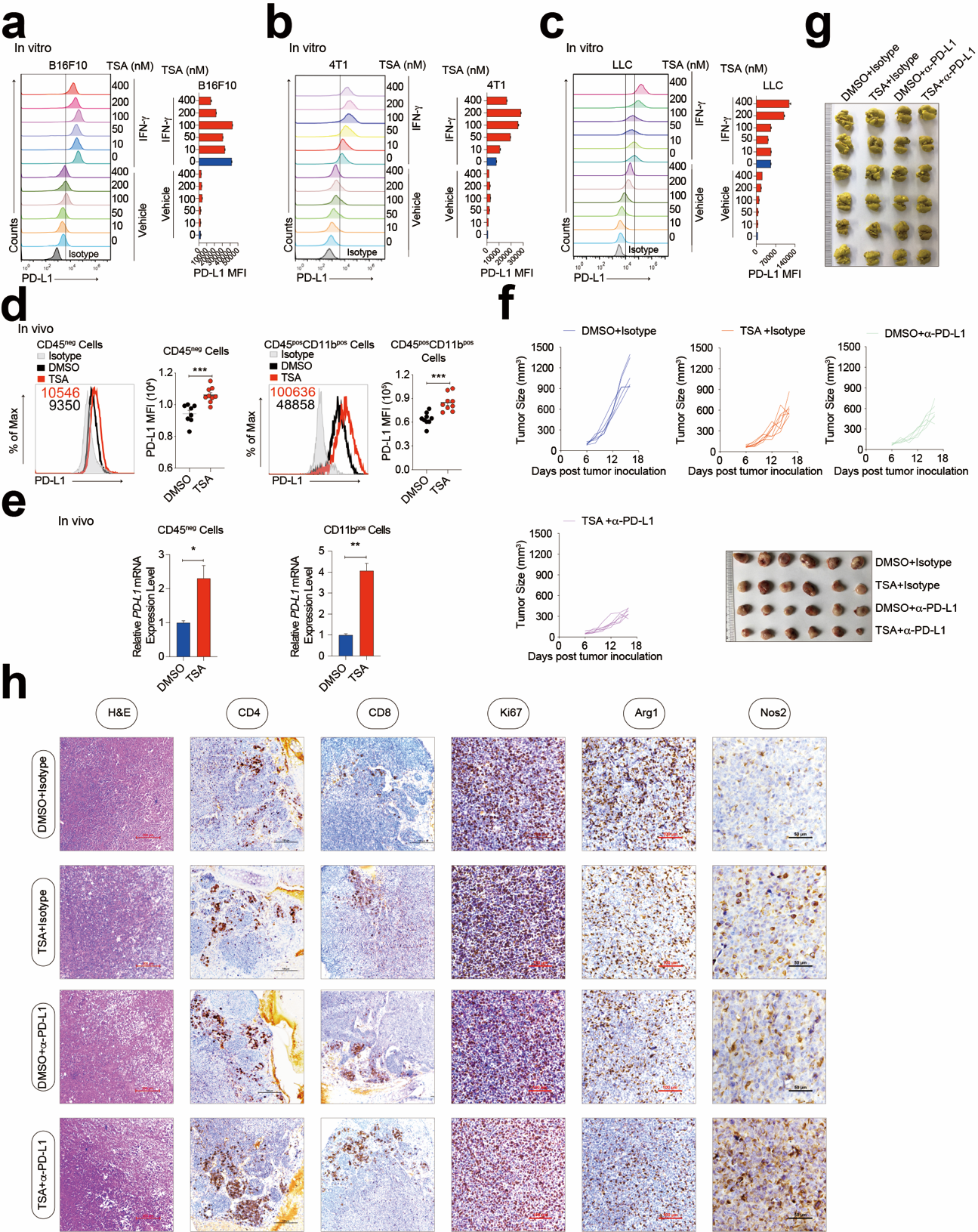
**

Supplement: Supplementary file 10 — Supplementary Figure 7 [file 41388_2020_1636_MOESM10_ESM.docx]
